# Autonomous robotic exploration with simultaneous environment and traversability models learning

**DOI:** 10.3389/frobt.2022.910113

**Published:** 2022-10-05

**Authors:** Miloš Prágr, Jan Bayer, Jan Faigl

**Affiliations:** Computational Robotics Laboratory, Faculty of Electrical Engineering, Czech Technical University in Prague, Prague, Czechia

**Keywords:** mobile robot exploration, active learning, traversability, multi-legged robot, locomotion gait

## Abstract

In this study, we address generalized autonomous mobile robot exploration of unknown environments where a robotic agent learns a traversability model and builds a spatial model of the environment. The agent can benefit from the model learned online in distinguishing what terrains are easy to traverse and which should be avoided. The proposed solution enables the learning of multiple traversability models, each associated with a particular locomotion gait, a walking pattern of a multi-legged walking robot. We propose to address the simultaneous learning of the environment and traversability models by a decoupled approach. Thus, navigation waypoints are generated using the current spatial and traversability models to gain the information necessary to improve the particular model during the robot’s motion in the environment. From the set of possible waypoints, the decision on where to navigate next is made based on the solution of the generalized traveling salesman problem that allows taking into account a planning horizon longer than a single myopic decision. The proposed approach has been verified in simulated scenarios and experimental deployments with a real hexapod walking robot with two locomotion gaits, suitable for different terrains. Based on the achieved results, the proposed method exploits the online learned traversability models and further supports the selection of the most appropriate locomotion gait for the particular terrain types.

## 1 Introduction

**FIGURE 1:**
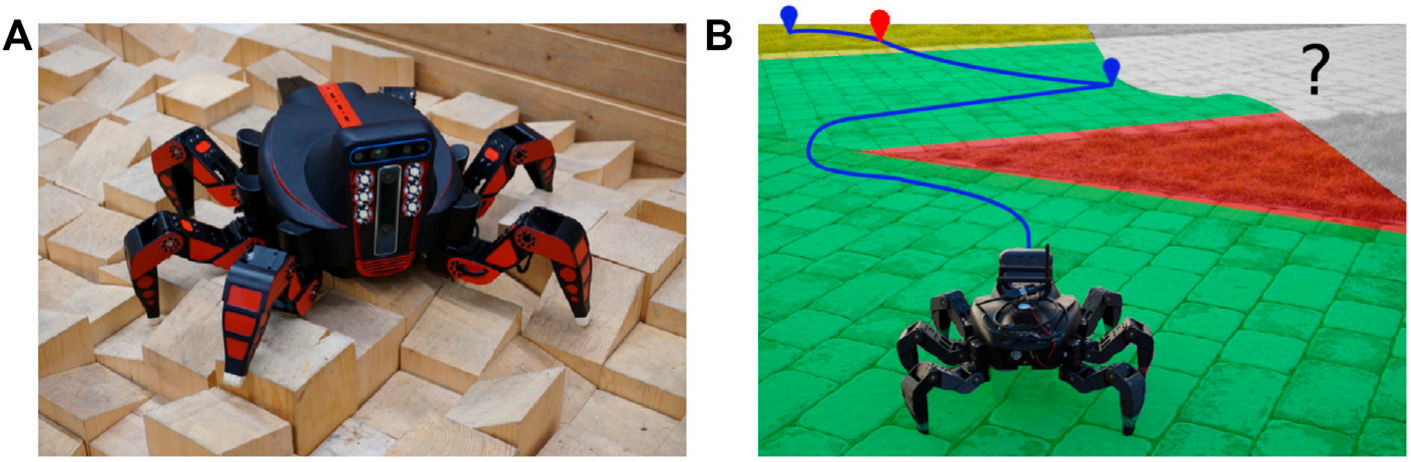
**(A)** Hexapod walking robot (courtesy of Forouhar et al. (2021)) **(B)** and its deployment using the proposed approach. The visualized planned path is to visit determined exploration goals for the spatial (in blue) and traversal cost models (in red). The spatial exploration goals are located close to the boundary of the already explored part of the environment. The traversal cost exploration goals correspond to sites where the terrain traversal cost model can be improved. Since the cost model is already partially learned, the red-tinted turf is known to be hard to traverse, and thus the robot prefers the green-tinted pavement, which is relatively easy to traverse. The yellow-tinted terrain is yet to be experienced by the robot and thus carries the terrain learning goal indicated by the red waypoint. The not-yet-observed area is gray.

The presented online terrain learning approach is motivated by long-term missions where autonomous robots would improve their operational performance in navigating *a priori* unknown environments. Some difficult to traverse terrains, such as large rocks, can be identified as obstacles using an observed geometric model of the environment. However, areas which appear flat and thus easy to traverse may, in practice, be hard to traverse due to their terra-mechanical properties, as experienced by NASA’s Mars Rover Spirit stuck in soft sand (Brown and Webster, 2010). In the presented approach, individual terra-mechanical properties are assumed to be partially unknown, and we learn a black box model to assess the traversability in a particular environment from the terrain appearance (Prágr et al., 2018). Since the scope of the functional relation between the terrain appearance and traversability might be limited to a particular environment, we advocate that on long-term deployments and exploration missions, the terrain models are learned online incrementally (Prágr et al., 2019b) as a part of the mission (Prágr et al., 2019a). Hence, we focus on the exploration of the environment and its terra-mechanical properties represented as the traversal costs that characterize the difficulty of traversing the individual terrains, as visualized in Figure 1. In particular, we consider multi-legged walking robots that can traverse various terrains with different traversal costs (also depending on the particular locomotion gait used), which provide a representative case for demonstrating the benefits of traversability assessment learned online. Compared to the previous work, the presented approach addresses the different locomotion gaits of the robot and distinguishes individual terrain-gait traversal cost models. In addition, the proposed exploration strategy provides a non-myopic (Zlot and Stentz, 2006) solution that takes into account both the spatial exploration and learning of the traversal cost models.

In the proposed approach, the impassable parts of the explored environment are determined by the geometric models using a grid-based elevation map (Bayer and Faigl, 2019). The individual terrain-gait traversal cost models are near-to-far predictors that infer the time to traverse over the traversable areas from their appearance and are learned using the robot’s previous experience accrued when traversing similar-appearing terrains using a particular gait. The traversal cost models comprise Gaussian process (GP) regressors (Rasmussen and Williams, 2006), which predict the traversal costs from the terrain appearance, and growing neural gas (GNG) (Fritzke, 1994) terrain type clustering schemes used to identify similar-appearing terrains. The geometric and traversal cost models are incrementally constructed while exploring the mission environment. The geometric model is continually built from the robot’s exteroception, whereas each traversal cost model accumulates the costs experienced by the robot when moving using the respective locomotion gait. During the deployment, each model continually provides a set of exploration goals to be visited to learn (improve) the model. For several possible goal locations, the exploration strategy is to determine a sequence of the navigational goals to be visited that is addressed as a solution of the *Generalized Traveling Salesman Problem* (GTSP) (Noon, 1988) to provide a non-myopic solution considering the so-called TSP distance cost (Faigl and Kulich, 2013).

The remainder of the article is organized as follows. In Section 2, we present an overview of the related approaches in mobile robot exploration and traversability assessment. Section 3 formally defines the studied problem of mobile robot exploration with *a priori* unknown terrain traversal cost assessment. The proposed exploration with online traversal cost learning is presented in Section 4. Section 5 reports on the performed experimental results in simulations and real-world experimental deployments with a multi-legged robot controlled by two motion gaits. In Section 6, we discuss the strong points and limitations of the proposed approach. Section 7 concludes the study.

## 2 State of the art

This section presents an overview of works related to the proposed approach. First, we focus on the traversability assessment approaches. Then we survey mobile robot exploration and environment modeling.

### 2.1 Mobile robot traversability

Two main questions emerge when reasoning about robot traversability over terrains. First, can the terrain be safely traversed, or should it be avoided? Second, if the terrain is passable, how does it compare to other terrains, i.e., is it easier and safer to traverse? Note that for the sake of clarity, we further denote the binary (true/false) traversability, which determines whether an area is an impassable obstacle or passable terrain, as terrain passability. In contrast, the relative comparison of the traversal difficulty over passable terrains is denoted as assessing the traversal cost. The term traversability is used to describe the notion in general, including both the passability and traversal cost. A review of mobile robot traversability assessment methods can be found in Papadakis (2013), and an overview of learning-based methods for ground robot navigation is in Guastella and Muscato (2021). Hence, we focus on works relevant to how traversability is approached in this study.

The passability discrimination can be directly incorporated in mapping in the form of occupancy cell grids (Moravec and Elfes, 1985), Gaussian mixtures (O’Meadhra et al., 2019), GP models (O’Callaghan et al., 2009), or Hilbert maps (Ramos and Ott, 2016). The distinction of terrain passability can be understood as an instance of terrain classification, where terrains are assigned individual classes, and each class carries presumed terra-mechanical properties. For example, some classes can be considered hard-to-traverse vegetation or obstacles (Bradley et al., 2015). In addition to terrain classification, terrains can be assigned continuous values describing some observed terrain property such as roughness (Krüsi et al., 2016; Belter et al., 2019), slope (Stelzer et al., 2012), or step height (Homberger et al., 2016; Wermelinger et al., 2016). For continuous measures, passability can be based on thresholding the value, as in Stelzer et al. (2012), where the passability is determined by individually thresholding terrain slope, roughness, and step height. Moreover, classes may correspond to a particular robot configuration, such as in Haddeler et al. (2020), where the authors classify terrains into modes of wheeled-legged locomotion.

In instances where the terra-mechanical properties are unknown and thus terrains’ appearance and geometry features are not sufficient to determine their traversability, the traversability can be based on the robot’s prior experience with similar terrains. The experience-based measures can be derived from the robot proprioception and described using stability (McGhee and Frank, 1968; Lin and Song, 1993), slippage (Gonzalez and Iagnemma, 2018), vibrations (Bekhti and Kobayashi, 2016), velocity, or energy consumption (Kottege et al., 2015). The experience-based approaches describe the traversal cost only over passable terrains since the traversal is needed to acquire the robot experience. An exception worth mentioning is haptic sensing to determine obstacle passability (Baleia et al., 2015), which, however, still relies on the direct interaction of the robot with the terrain.

Since the experience-based approaches use on-location robot experience, they are difficult to use directly in path planning where it is necessary to evaluate terrain traversability from a distance using only exteroceptive measurements. Near-to-far approaches pair traversability indicators that can be observed only near the robot (such as proprioception or dense short-range measurements) with terrain appearance and geometry that can be observed from farther distances and thus learn to predict traversability from the long-range measurements. Sofman et al. (2006) incrementally learned the relation between dense laser-based features characterizing ground unit traversability and overhead features that can be used to assess traversability from aerial images, whereas Bekhti and Kobayashi (2016) learned to predict vibration-based traversability from terrain texture. Quann et al. (2020) proposed an energy traversal cost regressor considering both terrain position and appearance. In addition, Mayuku et al. (2021) proposed a self-supervised labeling approach for a near-to-far scenario, where vibration-based traversal cost is inferred from image data, and the self-supervised data gathering is based on identified terrain classes.

Following the approaches in the literature, we assume that terrain is rigid, and it is possible to distinguish passable terrain and non-traversable obstacles from the terrain geometry using a step height similar to Stelzer et al. (2012), or Wermelinger et al. (2016). Hence, this study focuses on modeling the traversal cost over the determined passable terrains. Moreover, we are motivated by the online cost assessment in mobile robot exploration, where the computational requirements are crucial. Therefore, we avoid high fidelity models, which besides being costly to compute also rely on plan execution with high precision (such as deterministic foothold placement), which might not be available in practice. The traversal cost is thus learned as a black box near-to-far model that uses terrain appearance to predict the time to traverse over terrains. Since the scope of the relation between the terrain appearance and traversability might be limited to a particular environment, we incrementally learn the cost predictor by sampling the robot’s experience with traversing individual terrains. Similar to the classification in Belter et al. (2019), a color histogram is selected as the terrain appearance descriptor because it is simple to compute and the histograms are sufficiently descriptive to capture multi-colored terrains. Furthermore, we consider locomotion gaits of the employed hexapod walking robot that are suitable for different terrains. Thus, the passable terrain is a terrain traversable by at least one gait, and obstacles are terrain parts that none of the gaits can traverse. We propose a decoupled approach that predicts the traversal cost for each gait independently, and the robot then selects the most cost-efficient gait for each terrain.

Regarding the existing methods, the proposed approach is closest to Haddeler et al. (2020), where modes of the wheeled-legged robot are switched. In addition, the proposed approach is also close to the self-supervised, near-to-far traversability-learning approach proposed by Mayuku et al. (2021). In that regard, the primary contribution of the proposed approach is the integration of active traversability learning in mobile robot exploration, where the robot plans a non-myopic path to improve both the spatial and traversal cost models learned online during the deployment.

### 2.2 Mobile robot exploration and environment modeling

Mobile robot exploration is an active perception problem that concerns behaviors where the robot seeks to build a model of *a priori* unknown environment. The exploration entails the robot seeking areas that are in some capacity unknown to construct a map of the environment. The exploration thus inherently combines localization, navigation, and planning (Schultz et al., 1999) to decide where the robot should go next. Steering the robot navigation to not-yet-observed areas yields frontier-based exploration (Yamauchi, 1997), where the frontiers represent boundaries between the observed traversable area and the unknown space represented on an occupancy grid (Moravec and Elfes, 1985). Recently, in the octree-based environment model, frontiers are represented as mesh faces with few neighbors (Azpúrna et al., 2021).

Bourgault et al. (2002) and Makarenko et al. (2002) exploit the probabilistic representation on such an occupancy evidence grid and navigate to maximize the approximated occupancy information gain. Charrow et al. (2015) proposed to use Cauchy–Schwarz quadratic mutual information to speed up the information gain computation. In addition, approaches that rely on non-grid-based representation for navigation, such as meshes and topological maps, may retain cell or voxel grids to quantify the information gain (Dang et al., 2020).

In addition to mapping, robots also build models of environment-underlying phenomena that can be temperature models (Luo and Sycara, 2018) or spread of gas (Rhodes et al., 2020). The environment phenomenon can be considered spatial, and the goal is thus to learn the mapping from the position in the environment to the value of the phenomenon. Furthermore, a spatiotemporal model can be considered (Ma et al., 2018) that would require repeatedly visiting particular areas to build the temporal model, which might be needed for changing environments (Krajník et al., 2017).

Spatial-based modeling can be considered as informative path planning (Singh et al., 2007), where the goal is to find the most informative path through the environment (Hollinger and Sukhatme, 2014) subject to a particular constraint such as the robot energy budget (Binney and Sukhatme, 2012). Informative path planning approaches can be broadly divided into myopic and non-myopic methods. The myopic methods are greedy and plan only with regard to the next goal, whereas non-myopic methods plan with a longer horizon. For example, in the context of frontier-based mobile robot exploration, seeking the closest frontier is myopic, contrary to path planning to visit all the representatives of the frontiers that is non-myopic (Faigl et al., 2012).

Like seeking frontiers in spatial exploration, the explorer learning an underlying model must actively locate sites to sample novel information. Hence, GP regressors (Rasmussen and Williams, 2006) are particularly suited for active learning because it is relatively straightforward to identify uncertain regions where the model should be improved. GP prediction uncertainty is characterized by the differential entropy of the predicted normal distribution, leading to the characterization of information gained by observing individual areas. However, in practice, directly computing the information gained by possible observations is not feasible due to the number of possible actions, especially for a long planning horizon. Hence, various approximations and sampling strategies have been proposed.

Pasolli and Melgani (2011) proposed to either directly seek the most uncertain samples signified by the highest prediction variance or to select areas that are the most remote in the feature space given the GP hyper-parameters. In Viseras et al. (2019), the robot selects paths with high average entropy per sampling to tradeoff informativeness and the number of samplings. Martin and Corke (2014) proposed to set the mean function of a GP traversal cost regressors to zero, thus motivating a robot to traverse unknown areas where the predictions are close to the zero mean. The *GP Upper Confidence Bound* (GP-UCB) (Srinivas et al., 2010) is an exploration–exploitation method that combines seeking the most uncertain areas with improving the model around the highest value. It can be used when the learner is interested in finding extreme values of the modeled phenomenon, such as temperature (Luo and Sycara, 2018; Shi et al., 2020). In addition, a depth-first variant of the *Monte Carlo Tree Search* (MCTS) to select anytime informative paths can be employed to consider both differential entropy and upper confidence bound to model sampling informativeness (Guerrero et al., 2021).

Karolj et al. (2020) computed a path to the closest spatial frontier that visits all local sampling locations for a magnetism model by solving the *Traveling Salesman Problem* (TSP) over the respective goal locations. In localization in mapping, Ossenkopf et al. (2019) note that occupancy information gained at an unknown location holds little value and thus weight the occupancy gains by a pose uncertainty (Vallvé and Andrade-Cetto, 2015). Hence, the explorer must address how to combine the occupancy and pose uncertainties. In Bourgault et al. (2002) and Stachniss et al. (2005), the total exploration utility is a linear combination of the occupancy uncertainty and the robot localization uncertainty represented using the differential entropy based on its position distribution. In Carrillo et al. (2018), it is argued that combining Shannon’s discrete and differential entropies is neither practical nor sound because the differential entropy is neither invariant under a change of variable nor dimensionally correct. Therefore, both quantities may differ significantly in value. Consequently, Carrillo et al. (2018) proposed to use the localization uncertainty to weigh the Rényi entropy (Rényi, 1961) of the occupancy grid.

Based on the literature review on exploration approaches, we propose to generalize the previous work (Prágr et al., 2019a) toward a non-myopic approach. The therein proposed method combines active learning of traversal cost over terrains with spatial exploration using a greedy approach. The approximated spatial information gains and cost models are derived from Shannon’s discrete and differential entropies, respectively. Considering the reasoning of Carrillo et al. (2018), we avoid a direct combination of these two values in this study. In addition, we aim to build a modular system that supports the learning of models that range from the spatial map and cost predictors used in this study to temperature and pollution models. Hence, instead of creating a combined information gain utility function using the Rényi entropy, which is suitable for the combination of a map and robot’s localization model used by Carrillo et al. (2018), we elect to use a policy that combines the spatial exploration and cost learning goals (and goals reported by any additional model), similarly to the approach proposed by Karolj et al. (2020).

However, unlike the therein-built magnetism model, a spatial GP, we assume that the terrain traversal cost correlates with the terrain appearance. Therefore, the GP regressor infers the cost from the terrain feature descriptors instead of the terrain location. Consequently, rather than terrains nearby, sampling the cost to traverse an unknown terrain primarily affects the predictions over similarly appearing terrains close in the feature space. The affected terrains are determined using a terrain clustering scheme. Incremental growing neural gas (IGNG) (Prudent and Ennaji, 2005) is used to continually construct the terrain class structure, in which each class is assigned traversal cost and sampling reward (information gain) based on the GP’s predictions. As a result, we model the computation of the goal visit sequence as an instance of the *Generalized TSP* (GTSP) (Noon, 1988) (also called the Set TSP), which is a variant of the TSP where nodes are grouped into mutually exclusive and exhaustive sets. The problem is then to visit each set instead of visiting each node. In the context of the proposed exploration approach, the individual nodes correspond to possible sampling locations, and the sets are either terrain classes extracted from the cost prediction model or places where the robot can observe areas unknown to the spatial model.

The problem of mobile robot exploration with traversal cost learning is defined in the next section, whereas the strengths and weak points of the proposed approach are further discussed in Section 6.

## 3 Problem specification

**FIGURE 2:**
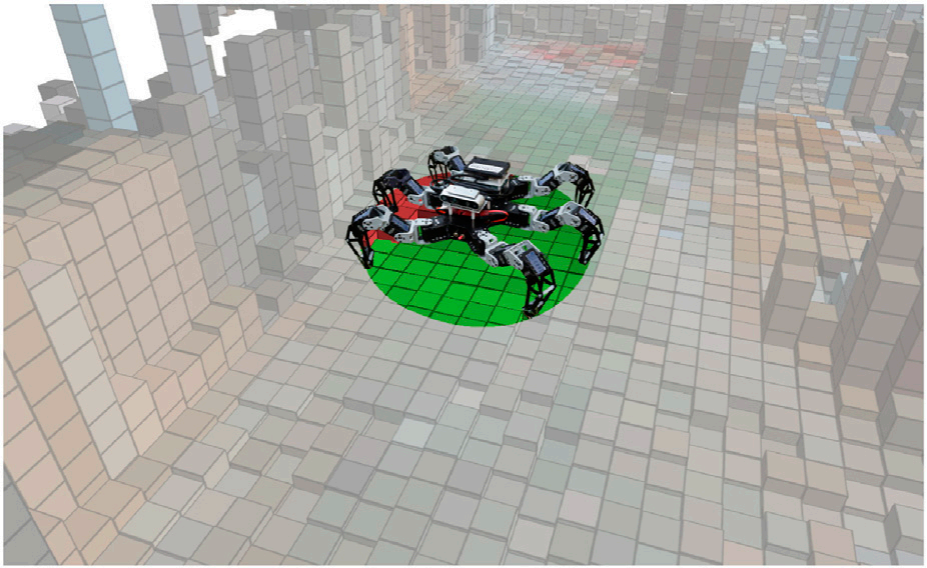
Footprint around the robot position covers the cells with potential multi-legged walking robot footholds.

The addressed exploration using an autonomous hexapod walking robot combines spatial exploration with active learning of terrain traversal cost models. The environment is modeled as a 2D grid $W\subset R^{2}$ with cells ν∈W with size *d*_*ν*_ corresponding to the size of the robot foothold. The position of the robot *p* ^robot^ is discretized as *ν* ^robot^ within the grid that is at the center of the robot’s circular footprint with radius *r*_robot_ covering all the potential robot’s footholds, as shown in Figure 2. Any path *ψ* can be decomposed to a sequence of neighboring cells as follows:

$$\begin{array}{cc} & \psi =\left(\nu_{1},\nu_{2},\ldots ,\nu_{n}\right),\\ \text{s.t.}\\ & \forall i\in 1,\ldots ,n:\;\pi \left(\nu_{i}\right)=1,\\ & \forall i\in 1,\ldots ,n-1:\;\nu_{i+1}\in 8nb\left(\nu_{i}\right), \end{array}$$ (1)

where *n* is the number of cells in the respective sequence, the function 8 nb(*ν*) lists the cells in the 8-neighborhood of *ν*, and *π*(*ν*) = 1 indicates that the cell *ν* is passable. In addition, the robot can use a discrete set of walking gaits G , and it is assumed that the gait changes occur instantaneously at the particular grid cells ν∈W .

The robot desires to move through the environment as efficiently as possible with respect to (w.r.t.) the cost *C*. Therefore, it moves along the cheapest path between *ν* and *ν*′.

$$\psi^{*}\left(\nu ,\nu^{\prime }\right)={\mathrm{argmin}}_{\psi \in \Psi \left(\nu ,\nu^{\prime }\right)}C\left(\psi \right),$$ (2)

where Ψ(*ν*, *ν*′) is the space of all paths from *ν* to *ν*′. The cost *C*(*ψ*) of traversing *ψ* represents a generic path cost such as time to traverse or expected consumed energy; without the loss of generality, the time to traverse is the cost of choice in this study. It is assumed that the cost is additive, thus permitting to combine the costs of two consecutive path segments *ψ*_*a*_ and *ψ*_*b*_ into the cost of the combined path *ψ*_*a*_ ⊕ *ψ*_*b*_ as follows:

$$C\left(\psi_{a}⊕\psi_{b}\right)=C\left(\psi_{a}\right)+C\left(\psi_{b}\right),$$ (3)

where ⊕ denotes the concatenation of the paths. The cost of a path is decomposed to the sequence of costs to traverse from passable cell *ν*_*a*_ to its neighbor *ν*_*b*_.

$$C\left(\psi \right)=\overset{n-1}{\underset{i=1}{\sum }}\|\left(\nu_{i},\nu_{i+1}\right)\|c\left(\nu_{i},\nu_{i+1}\right),$$ (4)

where ‖(*ν*_*a*_, *ν*_*b*_)‖ is the Euclidean distance between the cells (i.e., either *d*_*ν*_ or $\sqrt{2}d_{\nu }$ ), and *c* (*ν*_*a*_, *ν*_*b*_) is the per-meter cost of traversing from *ν*_*a*_ to *ν*_*b*_.

In the spatial exploration, the robot builds the geometry model P , which provides the cell passability assessment *π*(*ν*). It is assumed that the geometry is sufficient to distinguish the passable areas; hence, the passability model P is constructed directly from the continually streamed exteroceptive measurements (observed point clouds *z* ^pcd^).

### 3.1 Traversal cost modeling

The traversal cost is assumed to be too complex to be assessed only from the terrain geometry. In this study, the task is to learn a traversal cost predictor C that models the cost as a function of terrain appearance. The cost assessments are used in path planning w.r.t. (4). In addition, the cost model is also responsible for selecting the gaits suitable for the particular terrains traversed by the robot. Since the robot position is abstracted as the center of its circular footprint, the predictor’s per-meter-cost predictions are conservative estimates that take into account all the cells on the footprint. The cost predictor is learned online during the exploration from the robot experience, which comprises the cost *z* ^*c*^ experienced by the robot when traversing terrain described by the terrain appearance descriptor ta using gait *g*.

The learned model is compared to the uninformed baseline that represents a robot that only explores the spatial map and does not learn the cost models and thus uses the optimistic flat cost model.

$$\hat{c}\left(\nu_{a},\nu_{b}\right)=\frac{1}{v_{\text{max}}},$$ (5)

where *v*_max_ is the maximum robot velocity over all g∈G . Notice that, in planning, the particular value of *v*_max_ is not relevant as long as it is positive because it only scales the total cost, thus not affecting the planning decisions. The baseline selects the gaits reactively, using the fast gait capable of reaching *v*_max_ by default and switching to slower yet rough-terrain-capable gaits when the robot gets stuck on the traversed terrain.

The proposed approach is evaluated in model scenarios as follows. First, the robot is set to explore the environment W , and it incrementally learns the model C . Then the learned and baseline models are used in navigating the robot between a set of benchmark coordinates in W and the total cost *C* experienced by the robot (i.e., the time needed to move between the coordinates) using the particular model is considered to be the benchmark value.

## 4 Proposed system for active terrain learning in exploration

**FIGURE 3:**
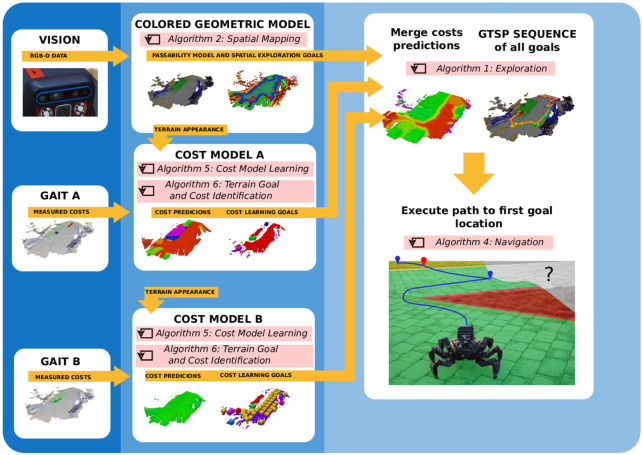
An overview of the proposed exploration system. The robot uses the RGB-D data to build the color elevation model of the environment in which it identifies the passable areas (Algorithm 2). The terrain appearance stored in the model is paired with the costs experienced by the robot to learn the traversal cost models for the individual locomotion gaits (Algorithm 5 and Algorithm 6). The cost predictions for the individual gaits and the terrain passability are used to plan the exploration path in a TSP sequence (Algorithm 1) over every goal reported by the geometric and cost models. The robot navigates to the first goal in the sequence (Algorithm 4).

In this section, we describe the proposed system for active terrain learning and exploration, which is overviewed in Figure 3. During the exploration, which yields the spatial geometric passability model P , the goal of the robot is also to learn the traversal cost model C . The geometric passability model P describes the shape of the environment and thus areas passable by the robot. The traversal cost model is decomposed into the set of models $C=C^{G}={\left\{C^{g}\right\}}_{g\in G}$ , where each traversal cost model $C^{g}$ predicts the costs associated with traversing the passable terrain using the gait g∈G . The respective cost predictors are Gaussian process (GP) regressors (Rasmussen and Williams, 2006), which use terrain appearance to infer the robot-experienced traversal cost accrued during the deployment. Each GP is coupled with the incremental growing neural gas (IGNG) (Prudent and Ennaji, 2005) that clusters similarly appearing terrains and hence identifies terrain types not yet visited by the robot. The exploration problem is modeled as an open-ended instance of the generalized traveling salesman problem (GTSP) (Noon, 1988), a variant of the TSP where the vertices are organized in disjoint sets, and each set is visited once. In this study, each set corresponds to an exploration or learning goal (a set of sampling sites) yielded by the spatial or cost model.

In the rest of the section, we describe the exploration process. The symbols used in the description are listed in Table 1. First, we show how the GTSP is used to find the exploration path. Then we show the geometric environment model in detail and the related passability model P , the traversal cost models $C^{g}$ , and their use to find the exploration goals.

**TABLE 1:** Used symbols.

Description	Symbol	Description	Symbol
World grid map model	W	Grid map cell	*ν*
Grid map cell size	*d*_*ν*_	Current robot position	*ν* ^robot^
Robot footprint radius	*r*_robot_	Cell *ν* passability	*π*(*ν*)
Path	*ψ*	Optimal path	*ψ*∗
Walking robot gait	*g*	Robot gait set	G
Cost (time to traverse)	*C*	Per-meter cost	*c*
Geometric passability model	P	Cost model	C
Measured cost	*z* ^*c*^	Maximum robot velocity	*v*_max_
Colored elevation grid map	$M_{2.5D}$	Robot sensor range	*r*_sensor_
Terrain appearance desciptor	ta	Descriptor radius	*r*_hist_
Spatial clustering radius	*c*_radius_	Cluster min cells	*c*_min cells_
Cost model, all gaits	$C^{G}$	Cost model, particular gait	$C^{g}$
Cost prediction, all gaits	$\hat{c}$	Cost prediction, particular gait	${\hat{c}}_{C^{g}}$
Distance transform per-meter loss	*c*_loss_	Cost measurement variance	$\sigma_{\text{sense}}^{2}$
Cost measurement filter initial variance	$\sigma_{0}^{2}$		
GP regressor	R	GP learning set	L
GP prediction mean	${\hat{\mu }}_{c}$	GP prediction variance	${{\hat{\sigma }}_{c}}^{2}$
Prediction uncertainty/GP entropy	*H*	High cost in cost transform	*c*_high_
Min learning set size	$n_{L}^{\text{min}}$	GP model noise variance	${\sigma_{\epsilon }}^{2}$
Exponential kernel length scale	*l*	Exponential kernel output variance	*σ*_*s*_
Maximum allowed cost	*c*_max_		
Terrain class model	T	Terrain class	*T*
Approximated cost information gain	$I_{C}$	Terrain class uncertainty threshold	$H_{C}^{\text{GT}}$
Min GT terrain type size	*m*_*T*_	Sampling lattice	*S*
Sampling lattice point	*p*_*S*_	Sampling lattice size	*d*_*S*_
Goal set	Γ	Goal	*γ*
Passability goal set	$\Gamma_{P}$	Cost goal set, all gaits	${\Gamma_{C}}^{G}$
Cost goal set, particular gait	${\Gamma_{C}}^{g}$	TSP distance matrix	*D*
Current exploration goal	$\nu_{E}^{*}$	Current exploration path	*ψ*_*E*_
Enforced sampling gait	*g* ^enforced^	Gait sampling duration	Δ*t*_sample_
IGNG structure	Ω	IGNG measurement	*x*
IGNG neuron set	*Ω*_neurons_	IGNG connection set	*Ω*_connections_
IGNG neuron	*ω*	IGNG adaptation threshold	*σ* ^IGNG^
IGNG winner warp rate	$\epsilon_{1}^{\text{IGNG}}$	IGNG neighbor warp rate	$\epsilon_{\text{nb}}^{\text{IGNG}}$
IGNG neuron mature age	$a_{\text{mature}}^{\text{IGNG}}$	IGNG connection maximum age	$a_{\text{max}}^{\text{IGNG}}$
Terrain type erosion steps	$n_{\text{erode}}^{\text{steps}}$	Terrain type dilation steps	$n_{\text{dilate}}^{\text{steps}}$
Terrain type dilation size	$n_{\text{dilate}}^{\text{size}}$		

### 4.1 Exploration

The robot explores the passability model P and learns the traversal cost models $C^{G}$ by visiting the exploration $\Gamma_{P}$ and cost learning ${\Gamma_{C}}^{G}$ goals, which are continually yielded by the respective models. Each goal $\gamma \in \Gamma_{P}\cup {\Gamma_{C}}^{G}$ is associated with a set of sites (cells) $\gamma ={\left\{\nu_{i}\right\}}_{i=0}^{\left|\gamma \right|}$ where the robot can improve its models by sampling the respective goal. The robot needs to visit one of the corresponding locations to sample the goal. Geometric model goals $\gamma \in \Gamma_{P}$ are located at singular sites *γ* = {*ν*}, where the robot can improve the spatial model by observing new areas. Each traversal cost model goal $\gamma \in {\Gamma_{C}}^{G}$ , where ${\Gamma_{C}}^{G}=\cup_{g\in G}{\Gamma_{C}}^{g}$ , is associated with a set of sites $\gamma ={\left\{\nu_{i}\right\}}_{i=0}^{\left|\gamma \right|}$ at which the robot can improve the model by experiencing novel gait-terrain costs. The areas covered by the individual goals in a given cost model are designed to be disjoint. Thus, sampling the traversal cost model at a site corresponding to the ${goal\;}^{2}{\gamma_{C}}^{g}\in {\Gamma_{C}}^{g}$ provides no, or severely limited, information regarding the traversal cost model at a site corresponding to a different ${goal}^{1}{\gamma_{C}}^{g}\neq^{1}{\gamma_{C}}^{g}$ . On the other hand, the passability and traversal cost models are considered independent. Sampling at one particular site might improve both models since the robot can observe previously unseen areas while experiencing untraversed terrain. However, two cost models cannot be improved at once since the robot can only experience the cost for the currently used gait.

Given the current robot position $\nu_{t}^{\text{robot}}$ and models $P_{t}$ and $C_{t}^{G}$ at any time *t* during the exploration, the robot selects a shortest exploration path $\psi_{E}\left(p_{t}^{\text{robot}},P_{t},C_{t}^{G}\right)$ that visits at least one site corresponding to each goal. The path planning is modeled as an instance of the GTSP, where vertices (sites) are organized in disjoint sets (goals), and each set is visited exactly once. The distance matrix *D* describes the costs of paths between the individual sites, including the distances between the current robot position and the goal sites.

$$D\left(\nu ,\nu^{\prime }\right)=\hat{C}\left(\psi^{*}\left(\nu ,\nu^{\prime }\right)\right).$$ (6)

**FIGURE 4:**
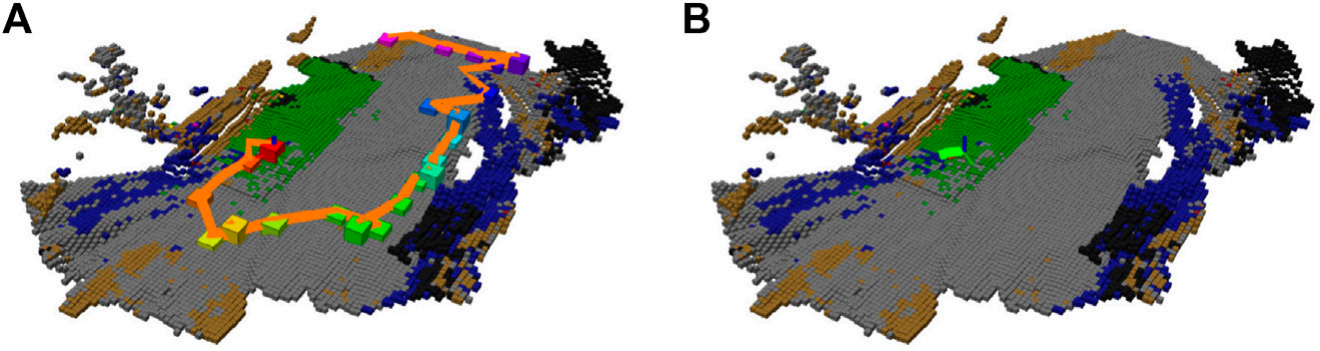
An example of a planned exploration path. **(A)** Global path over the sequence of goals determined by the TSP solver; **(B)** the local path to the first goal.

A total of two transforms are applied to the distance matrix *D* to create an open instance of the GTSP. First, the robot does not need to return to its current position after exploring the environment. Hence, the problem is transformed by setting the cost to reach the current robot position from any goal as zero $\forall \gamma \in \Gamma_{P}\cup \Gamma_{C}^{G},\forall_{\nu }\in \gamma :D\left(\nu_{\gamma },\nu^{\text{robot}}\right)=0$ . Second, we apply the Noon–Bean transformation (Noon and Bean, 1993) to transform an instance of the GTSP into an instance of the TSP. The open instances of the transformed TSP are solved by the LKH solver (Helsgaun, 2000), a heuristic solver with asymptotic time complexity bounded by $O\left(m^{2.2}\right)$ , where *m* is the number of vertices, which has been found sufficient for updates with tens of goal sites. The solver returns the sequence of sites (*ν* ^robot^, *ν*_0_, *ν*_1_, … , *ν*_*n*_) to be visited through the environment, see Figure 4A, where *n* is the number of goals and each site *ν*_*i*_ corresponds to a different goal. The robot navigates toward the first site of the sequence and its current exploration goal $\nu_{E}^{*}$ becomes $\nu_{E}^{*}=\nu_{0}$ , see an example of the path in Figure 4B.

The plan is recomputed on-demand either when there is a change in the goal set or as a result of reaching the current goal. Moreover, upon reaching a cost model goal, the robot switches to the model’s respective gait *g* ^enforced^ and is forced to move forward for Δ*t*_sample_ (or until an obstacle is reached) to sample the traversal cost over the terrain. The exploration ends when every model reports zero goals. The exploration process is summarized in Algorithm 1.

Algorithm 1 Exploration.

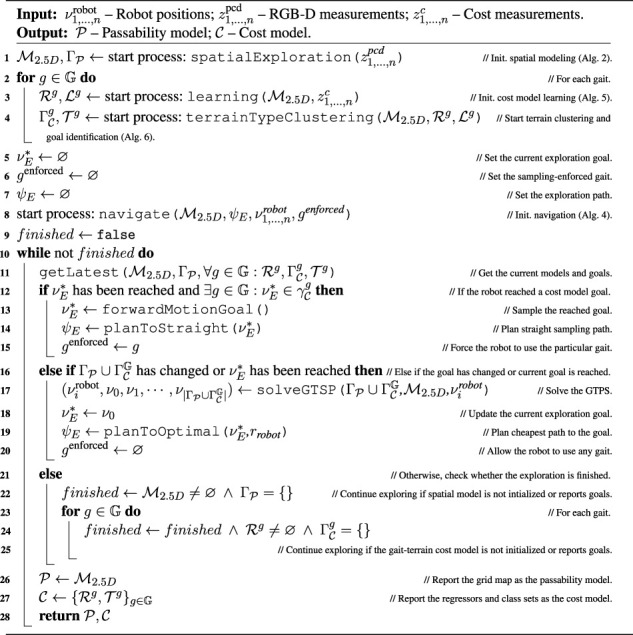

### 4.2 Environment geometry & passability model

The grid environment W is represented by the colored elevation grid map $M_{2.5D}$ with the cell size *d*_*ν*_. The grid map is built online during the exploration according to Algorithm 2 using the robot’s range measurements and RGB camera images. The elevation at each cell $\nu \in M_{2.5D}$ is obtained by fusing the localized range measurements $z_{i}^{\mathrm{pcd}}$ into the grid map using an one dimensional Kalman filter, as described in Fankhauser et al. (2014) or Bayer and Faigl (2020). The localization of the robot, and also the localization of the range measurements, is considered to be solved by the Intel RealSense T265 tracking camera, which estimates the robot’s full six DOF pose based on visual simultaneous localization and mapping supported by an inbuilt inertial measurement unit^1^. The grid map is used as a model of the terrain geometry to identify passable places. It also captures the color of the terrain texture that is processed to compute the terrain appearance descriptors.

Algorithm 2 Spatial exploration.

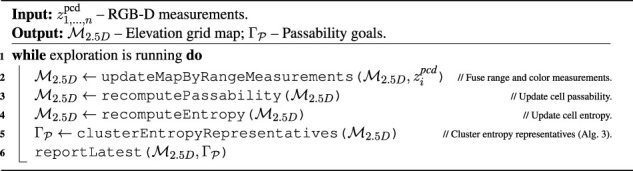

**FIGURE 5:**
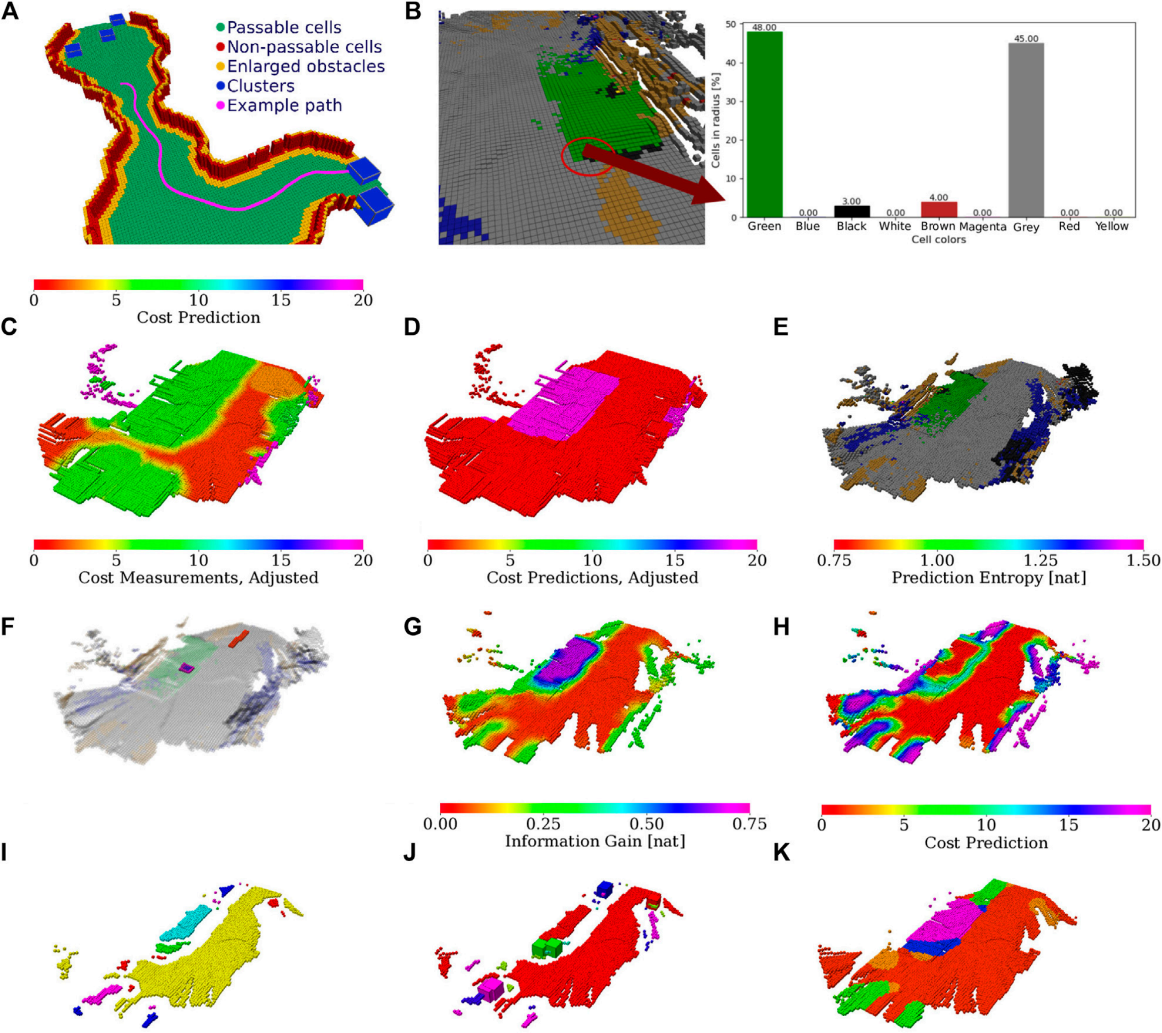
Illustration of the color-geometric and cost models. **(A)** Visualization of the online built geometrical model with marked passability and clusters based on the cells with non-zero information according to the shown color legend; **(B)** terrain appearance descriptor calculated as a histogram of cell colors. The costs used in path planning; **(C)** minimal cost over gaits after the distance transform; **(D)** respective cheapest gait (gaits in red and purple). **(E)** Colors used to build the color histogram terrain appearance descriptor; **(F)** measured costs used for learning the GP (adjusted by hyperbolic tangent), visualized over the terrain appearance; **(G)** raw GP cost prediction; **(H)** GP prediction uncertainty. **(I)** Terrain clusters (arbitrary colors used to distinguish clusters); **(J)** information gained with terrain learning goals (goal colors corresponding to clusters); **(K)** cluster costs used in planning.

We define the passability of the cell $\nu \in M_{2.5D}$ as the probability *π*(*ν*) that the cell *ν* can be traversed by the robot. The probability itself is based on the observed roughness of the terrain computed based on Bayer and Faigl (2021) as follows:

$$\rho \left(\nu \right)=\underset{\nu^{\prime }\in 8nb\left(\nu \right)}{\max}\Delta \left(\nu ,\nu^{\prime }\right),$$ (7)

where 8 nb(*ν*) is the 8-neighborhood of the cell *ν*, and the step height Δ(*ν*_*a*_, *ν*_*b*_) is as follows:

$$\Delta \left(\nu_{a},\nu_{b}\right)=|elevation\left(\nu_{a}\right)-elevation\left(\nu_{b}\right)|,$$ (8)

where elevation(*ν*) denotes the estimated height of the terrain at *ν*. The probability that the robot can pass a cell *ν* is as follows:

$$\pi \left(\nu \right)\left\{\begin{array}{cc} 0\; & \text{if }\rho \left(\nu \right)>\rho_{\text{obstacle}}\\ 1\; & \text{otherwise} \end{array}\right.,$$ (9)

where the threshold *ρ*_obstacle_ represents the lowest obstacle to be detected. An example of the grid map is shown in Figure 5A.

In active perception scenarios, the information about the terrain model $M_{2.5D}$ gained by observing the cell *ν*′ is evaluated by entropy based on the known passability. Since the distribution of the passability is binary and depends on the 8-neighborhood of the cell, information gained by observing *ν*′ with unknown height is approximated as follows:

$${I_{P}}^{\text{cell}}\left(\nu^{\prime }\right)\approx \frac{k\left(\nu^{\prime }\right)+1}{9},$$ (10)

where *k*(*ν*) is the number of the unknown cells in the neighborhood of *ν*. Thus, the expected information gained by perceiving the terrain from the position of the cell *ν* can be expressed as follows:

$${I_{P}}^{\text{model}}\left(\nu \right)=\underset{\nu^{\prime }\in \delta \left(r_{\text{sensor}},\nu \right)}{\sum }\left\{\begin{array}{cc} {I_{P}}^{\text{cell}}\left(\nu^{\prime }\right)\; & \text{if }\;observable\left(\nu ,\nu^{\prime }\right)\\ 0\; & \text{otherwise} \end{array}\right.,$$ (11)

where *δ*(*r*_sensor_, *ν*) is the sensor range *r*_sensor_-large neighborhood of *ν*; the function observable (*ν*, *ν*′) returns true if and only if the cell *ν*′ is observable from *ν*, which is determined by casting a ray from *ν* to *ν*′ in the current elevation map $M_{2.5D}$ . Using all the cells with non-zero entropy in the TSP formulation is computationally intensive. Thus, we propose to spatially cluster the entropy to generate a limited number of spatial entropy representants by Algorithm 3.

Algorithm 3 Cluster entropy representatives

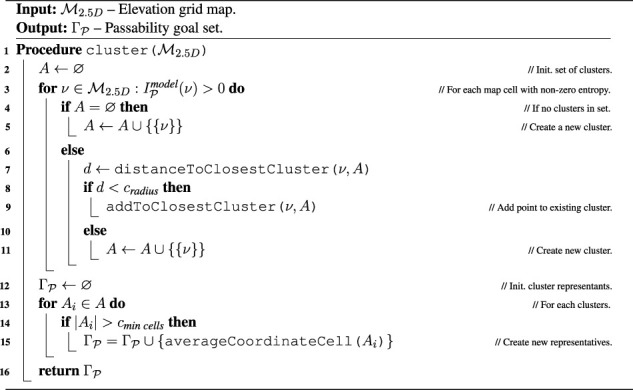

In addition to the terrain geometry, the grid map $M_{2.5D}$ also carries the terrain texture calculated by the following approach. Each cell is provided a 10-bit color by projecting the camera image to the map $M_{2.5D}$ . Then the color space is shrunk to nine different colors, defined by color prototypes listed in Figure 5B. The relative amount of the cell colors within the radius *r*_hist_ matched to the selected color prototypes are used to build a 9-dimensional terrain appearance descriptor ta(*ν*) for each cell $\nu \in M_{2.5D}$ , which is visualized as a color histogram in Figure 5B.

### 4.3 Traversal cost model

The cost model C predicts the per-meter traversal cost *c* over observed areas deemed passable by the geometric passability model P . The traversal cost model predicts the traversal cost from terrain appearance. Since the robot position is abstracted as the center of its circular footprint, the C ’s per-meter-cost predictions are conservative estimates that take into account all the cells on the footprint.

$$\hat{c}\left(\nu_{a},\nu_{b}\right)={max}_{\nu^{\prime }\in \delta \left(r_{\text{robot}},\nu_{a}\right)}\hat{c}\left(\nu^{\prime }\right),$$ (12)

where *δ*(*r*, *ν*) lists all cells within the *r*-radius of cell *ν*, and $\hat{c}\left(\nu \right)$ is the C cost estimate over cell *ν*. An example of the traversal cost assessment is depicted in Figure 5C.

The cost $\hat{c}$ is reported for the whole model set $C=C^{G}={\left\{C^{g}\right\}}_{g\in G}$ , since it is the best gait-terrain cost.

$$\hat{c}\left(\nu \right)={min}_{g\in G}{\hat{c}}_{C^{g}}\left(\nu \right),$$ (13)

where each gait-terrain cost ${\hat{c}}_{C^{g}}$ is the prediction of the particular model $C^{g}$ . In addition, when navigating through the environment, the robot selects its gait w.r.t. the minimization in Eq. 13, as depicted in Algorithm 4. An example of gait selection is visualized in Figure 5D. A distance transform with *c*_loss_ per-meter-loss is used over the cell grid with the best-gait costs $\hat{c}\left(\nu \right)$ to dissuade the robot from navigating areas near terrain boundaries where frequent gait changes are likely.

Algorithm 4 Navigate

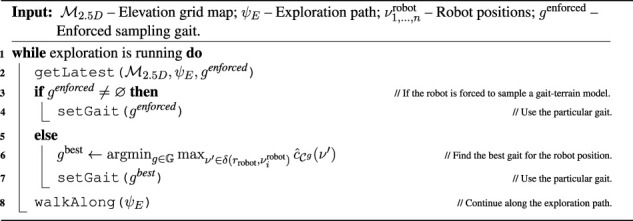

Each gait-terrain model $C^{g}$ comprises the cost regressor R and the terrain type clustering T . In R , we use GP regression to predict the traversal costs because it provides the predicted values and models the prediction uncertainty. Each traversal cost regressor R is learned from the learning set L of the paired terrain descriptors and the respective traversal costs observed when using the particular gait *g* that are depicted in Figure 5E and Figure 5F, respectively. The particular learned cost regressor R is used to predict the normal distribution of the traversal cost at queried terrain descriptor ta as follows:

$$N\left({\hat{\mu }}_{c},{{\hat{\sigma }}_{c}}^{2}\right)\left(ta,R\right)=predict\left(ta,R\right).$$ (14)

The cost prediction (visualized in Figure 5G) is the expected value.

$$\hat{c}\left(ta,R\right)=E\left(N\left({\hat{\mu }}_{c},{{\hat{\sigma }}_{c}}^{2}\right)\left(ta,R\right)\right)={\hat{\mu }}_{c}\left(ta,R\right),$$ (15)

and the uncertainty of the prediction (shown in Figure 5H) is characterized by the differential entropy.

$$H\left(N\left({\hat{\mu }}_{c},{{\hat{\sigma }}_{c}}^{2}\right)\left(ta,R\right)\right)=\frac{1}{2}\log\left(2\pi e{{\hat{\sigma }}_{c}}^{2}\left(ta,R\right)\right).$$ (16)

The prediction uncertainty is used to approximate the information gain $I_{C}$ associated with sampling the individual observed terrains, thus identifying areas the robot needs to visit to improve the traversal cost model.

The terrain type clustering T identifies the distinct terrain types (terrain descriptor clusters) in the environment. The terrain class set T is designed to be disjoint regarding the prediction model. Thus, sampling the traversal cost model at a cell corresponding to one terrain class provides no, or severely limited, information regarding the traversal cost model at a location corresponding to a different class. In particular, following Pasolli and Melgani (2011), the classes are selected to be mutually distant in the terrain descriptor space. Each observed cell is assigned the closest terrain class as the closest class in the descriptor space.

$$T^{*}\left(\nu \right)={\mathrm{argmin}}_{T\in T}\|ta\left(\nu \right),ta\left(T\right)\|,$$ (17)

where ta(*T*) is the appearance assigned to the terrain class T∈T . Since, on small terrain classes, it might not be possible to acquire enough samples to learn the traversal cost with sufficient certainty, we apply class erosion as described in Supplementary Appendix S1. The erosion output is the learning class assignment *T* and the planning class assignment $\hat{T}$ . We avoid computing the cost prediction for every cell independently^2^ and report the $C^{g}$ prediction over a particular area as the cost to traverse over its respective terrain type.

$${\hat{c}}_{C^{g}}\left(\nu \right)=\left\{\begin{array}{cc} \hat{c}\left(ta\left(\hat{T}\left(\nu \right)\right),R\right)\; & \text{if}\;\hat{T}\left(\nu \right)\neq \emptyset ,\\ c_{\text{max}}\; & \text{otherwise}, \end{array}\right.$$ (18)

where the maximum cost *c*_max_ is reported for cells with no class (i.e., eroded) *∅*.

The rest of this section describes how the traversal cost experience used to learn the models is measured, how the GP regressor is learned, and how the terrain type clustering is used to identify the locations where to improve the cost model.

### 4.3.1 Traversal cost measurement

The measured traversal cost describes the time needed to traverse between cells as *z* ^*c*^ (*ν*, *ν*′). Since the distance between 2 cells is significantly lower than the robot stride length, the cost is smoothed over path segments (cell sequences) with a fixed duration. In particular, the per-meter cost *c* is continually measured as the inverted robot velocity *v* ^−1^ over the path segment traversed by the robot in the last Δ *t* s.

$$v^{-1}\left(\psi_{s}\right)=\frac{T\left(\psi_{s}\right)}{\left\|\psi_{s}\right\|},$$ (19)

where ‖*ψ*_*s*_‖ is the length of the segment in meters and *T*(*ψ*) is the measurement duration that is fixed to Δ*t*. If the robot had not changed its gait on the segment, the cost is reported to the particular model $C^{g}$ as the cost to traverse the midpoint of the segment as $z^{c}\left(\nu_{\left\lfloor |\psi_{s}|/2\right\rfloor },\nu_{\left\lfloor |\psi_{s}|/2\right\rfloor +1}\right)$ . In addition, to remove potential cost spikes, the cost is further smoothed using a moving average window of the same (Δ*t*) duration. Since the inverse velocity is unbounded and has both high values and high variance for a stuck robot, the cost to be used by the predictor is transformed as follows:

$$c=c_{\text{high}}\tanh\left(\frac{1}{c_{\text{high}}}\frac{v^{-1}}{{v_{\text{max}}}^{-1}}\right),$$ (20)

where the maximum robot velocity *v*_max_ (maximum from all g∈G ) scales the cost of the robot moving over an ideal terrain to 1, and the high cost *c*_high_, which should only be experienced by a stuck robot, is used in the transform to bound the cost values.

### 4.3.2 Gaussian process traversal cost regressor

The employed GP regressor predicts both the prediction mean and variance making it suitable to model the prediction distribution as in (Eq. 14). Its description is dedicated to Supplementary Appendix S2 to make the study self-contained. GP regressor is learned only if there are at least $n_{L}^{\text{min}}$ learning pairs in L to avoid learning overconfident predictors at the beginning of the exploration. The learning is summarized in Algorithm 5.

Algorithm 5 Traversal cost model learning.

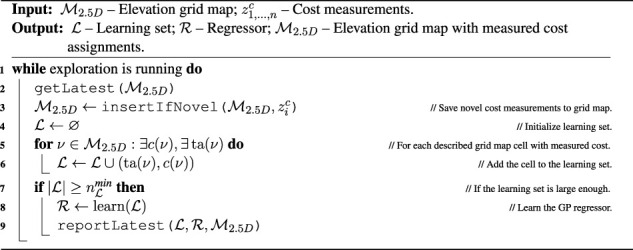

The covariance function used in this work is the squared exponential kernel.

$$K\left(x,x^{\prime }\right)={\sigma_{s}}^{2}\exp\left(-\frac{1}{2}\frac{{\left(x-x^{\prime }\right)}^{2}}{l^{2}}\right),$$ (21)

where ${\sigma_{s}}^{2}$ is the output variance, and *l* is the length scale. We consider that the robot’s cost and feature models have known ranges based on (Eq. 20) and the histogram descriptor, respectively. Therefore, similar to Karolj et al. (2020), the kernel hyperparameters *l* and ${\sigma_{s}}^{2}$ and GP’s ${\sigma_{\epsilon }}^{2}$ have fixed values that we consider to be dependent on the system parameters.

The GP is continually relearned when new observations using the particular gait *g* are experienced. The learning complexity can be bounded by $O\left(n^{4}\right)$ , where *n* is the number of training points. The size of the learning set L is limited by using at most one training point corresponding to each cell in $M_{2.5D}$ and by storing measurements only when they are novel and thus likely to improve the model. Hence, the relative traversal cost *c*(*ν*) experienced at cell *ν* is paired with the appearance descriptor ta(*ν*) of the respective traversed terrain, and when building the learning set L , the model reports the pair (ta(*ν*), *c*(*ν*)) for each cell where both values are available.

Since the robot keeps only one measurement for each cell, each novel cost measurement *z* ^*c*^ (*ν*, *ν*′) experienced when using the gait *g* is allocated to the grid map cell *ν* and its neighbors in 8 nb(*ν*), and the traversal cost *c*(*ν*) at the cell *ν* is modeled using the Kalman filter with the estimated value and covariance as follows:

$$c_{k}=\frac{\sigma_{\text{sense}}^{2}c_{k-1}+\sigma_{k-1}^{2}{z^{c}}_{k}}{\sigma_{\text{sense}}^{2}+\sigma_{k-1}^{2}},\;\sigma_{k}^{2}=\frac{\sigma_{\text{sense}}^{2}\sigma_{k-1}^{2}}{\sigma_{\text{sense}}^{2}+\sigma_{k-1}^{2}},$$ (22)

where ${z^{c}}_{k}$ is the *k*th cost measurement at *ν* and $\sigma_{\text{sense}}^{2}$ is its variance. The filter is initialized by the first cost observation ${z^{c}}_{0}$ at the respective cell, and the initial filter variance is $\sigma_{0}^{2}$ .

In total, two cases are considered as situations when the cost is novel, and thus the model should be improved by storing the cost w.r.t. (Eq. 22): 1) when the prediction is erroneous and 2) when the prediction is uncertain. For the former, the cost experienced at the cell *ν* is accumulated if the measured cost *z* ^*c*^ is out of the approximate 95% confidence interval $|{\hat{\mu }}_{c}\left(ta\left(\nu \right)\right)-z^{c}|>2{\hat{\sigma }}_{c}\left(ta\left(\nu \right)\right)$ of the prediction at *ν*. For the latter, the approximated information gain of the prediction is considered, and the robot accrues measurements when there is a potential of information gain $I_{C}\left(T\left(\nu \right)\right)>0$ , which computation is described in the following paragraphs.

### 4.3.3 Terrain type clustering and goal identification

The traversal cost exploration goals ${\Gamma_{C}}^{g}$ are selected by the robot as areas where the model can be improved and thus are the areas where the traversal cost model is uncertain. Each goal represents a terrain class where the robot can sample novel information about the cost model. The overall approach to goal identification is summarized in Algorithm 6.

Algorithm 6 Terrain type clustering, goal identification, and cost identification.

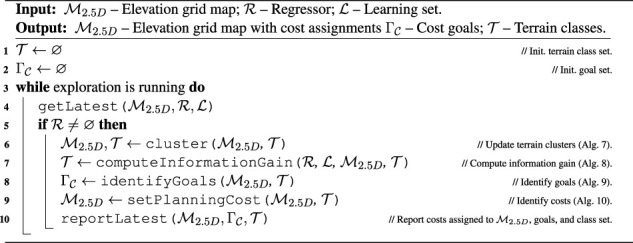

Algorithm 7 Cluster.

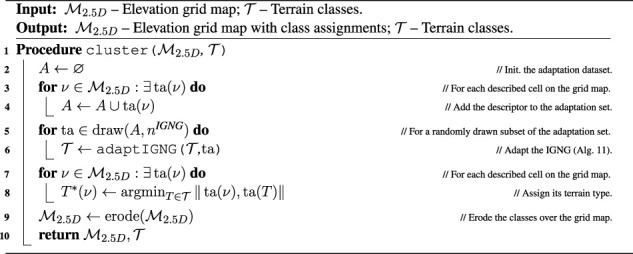

The clustering scheme presented in Algorithm 7 is based on the IGNG, described in Supplementary Algorithm S1, to make the study self-contained. In the neural gas, each neuron is a terrain prototype ta(*T*) in the descriptor space that represents a terrain class *T*. When separating the classes, the intuition is that for exponential kernels, the length scale describes the range from the data where the model can reliably extrapolate, as used, for example, in Karolj et al. (2020). Hence, new classes are inserted into the neural gas when the distance from all prototypes exceeds *σ* ^IGNG^ = 2*l*. The neural gas is constructed incrementally by repeated adaptation using the appearance descriptors in the environment, where the size of each adaptation batch is limited to *n* ^IGNG^ descriptors that are randomly sampled from all the descriptors, and the yielded terrain classes can be seen in Figure 5I.

Algorithm 8 Compute information gain.

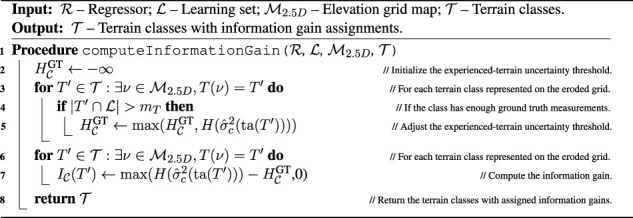

The terrain classes for which the cost model can be improved are identified using the cost regressor R -predicted traversal cost distribution $N\left({\hat{\mu }}_{c},{{\hat{\sigma }}_{c}}^{2}\right)\left(ta\left(T\right)\right)$ at the class prototypes ta(*T*). The traversal cost exploration goals are selected according to Algorithm 8 as the classes where there is potential for information gain; see the visualization in Figure 5J. The gain is approximated from the prediction entropy.

$$I_{C}\left(T\right)\approx max\left(H\left({{\hat{\sigma }}_{c}}^{2}\left(ta\left(T\right)\right)\right)-H_{C}^{\text{GT}}\left(L\right),0\right),$$ (23)

where $H_{C}^{\text{GT}}$ is a threshold value associated with the uncertainty of the experienced traversal costs. The robot learns when there is potential of information gain $I_{C}>0$ , and no information can be gained at eroded cells $I_{C}\left(\emptyset \right)=0$ . We set the threshold value based on the highest prediction uncertainty for terrains that are considered certain since they cover cells that are already in the learning set as follows:

$$H_{C}^{\text{GT}}\left(L\right)=\underset{T\in T:|\left\{\nu \in M_{2.5D}:T\left(\nu \right)=T\right\}\cap L|>m_{T}}{\max}H\left({{\hat{\sigma }}_{c}}^{2}\left(ta\left(T\right)\right)\right),$$ (24)

where we avoid overconfident GP-predictions for barely sampled terrains by allowing only terrain classes with at least *m*_*T*_ observed ground truth cost values. The threshold equals the maximum value over such ground truth terrain classes.

Algorithm 9 Identify goals.

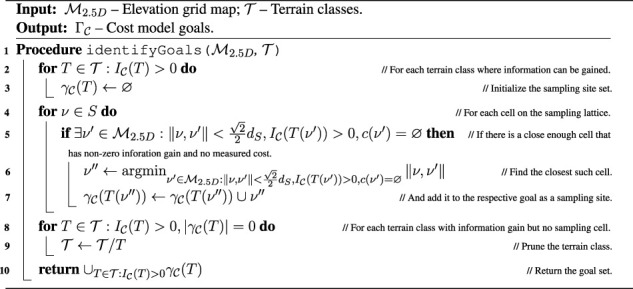

The sampling locations (visualized, for example, in Figure 5J) corresponding to the terrain class are sampled along a lattice *S* with the cell size *d*_*S*_ ≫ *d*_*ν*_, as depicted in Algorithm 9. For each lattice point *p*_*S*_, the closest cell *ν* in $\delta \left(\frac{\sqrt{2}d_{S}}{2},p_{S}\right)$ radius that is not associated with a traverability measurement and that is informative with $I_{C}\left(T\left(\nu \right)\right)>0$ is reported as a sampling site; if no such cell exists, no site is reported for the lattice point. Since only cells without measurements are considered, it is possible for small terrain classes to run out of cells before reaching *m*_*T*_ measurements. In such a case, the class is considered too small to learn and is no longer reported as a goal, and it is pruned from the class set. In addition to the goals, the traversal cost ${\hat{c}}_{C^{g}}\left(\nu \right)$ (visualized in Figure 5K) is also reported for the *ν*′s prototype $ta\left(\hat{T}\left(\nu \right)\right)$ w.r.t. (Eq. 13) according to Algorithm 10.

Algorithm 10 Set planning cost.

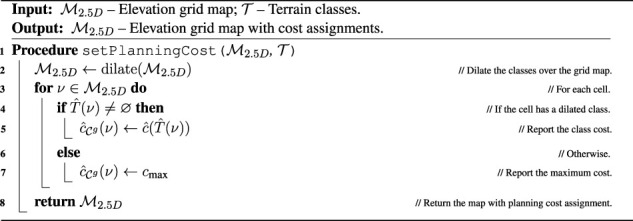

## 5 Experimental evaluation

The proposed exploration with active terrain learning has been examined in simulated trials and real experimental deployments using a hexapod walking robot. The simulated and real scenarios have been set up so that the robot first explores the environment and learns the cost models using the proposed method and, in some tests, a selected baseline method. Then the performance has been evaluated and compared with the baseline approach by navigating the robot over a sequence of benchmark waypoints using the respective traversal cost models of the environment learned during the exploration.

The hexapod walking robot, which can be seen in Figure 1, is used in the real deployment, and the simulation is parameterized to mimic the robot’s motion and sensory capabilities. The robot has six legs, each comprising three Dynamixel XM430-W350 servomotors. The robot is equipped with the Intel RealSense D435 camera used to construct the colored environment model and the Intel RealSense T265 localization camera. The onboard computation is provided by the Intel NUC 10i7FNK with Intel Core i7 10710U accompanied with 64 GB memory, running Ubuntu 18.04 with ROS Melodic (Quigley et al., 2009). The robot locomotion is facilitated by a blind adaptive motion gait (Faigl and Čížek, 2019). The robot uses two particular gait configurations, see Table 2: The fast gait suitable for flat, even surfaces, and the tall gait that performs better than the fast gait over rough terrain but otherwise is slower. The robot is equipped with a reflex that detects that the robot is stuck with costs exceeding *c*_max_ and switches over to the tall for Δ*t*_fallback_ seconds to avoid the robot getting stuck when using the baseline model or at the beginning of the learning process. The parameterization of the proposed method can be found in Table 3, and the operating frequencies of the proposed method’s processes are depicted in Table 4.

**TABLE 2:** Gait parameterization.

Gait parameter/gait	Fast gait	Tall gait
Gait cycle duration (s)	1.10	2.90
Step height (m)	0.04	0.07
Maximum forward speed (ms^−1^)	0.05	1.25*e* − 2

**TABLE 3:** System parameterization.

Symbol	Parameter	Unit	Value, split by environment	
			Real/Small Sim.	Large Sim.
*d*_*ν*_	Grid map cell size	m	0.05	0.10
*r*_sensor_	Sensor range	m	2.5	10.00
*c*_radius_	Spatial clustering radius	m	0.50	2.00
*c*_min _*cells*_	Spatial clustering, min cells per cluster	-	10	10
*r*_robot_	Robot footprint radius	m	0.25	0.40
*ρ*_obstacle_	Roughness passability threshold	m	0.25	0.25
*r*_hist_	Histogram descriptor radius	m	0.25	0.30
Δ*t*	Cost measurement window duration	s	5.00	1.00
*v*_max_	Maximum robot velocity	m s^−1^	0.05	0.25
*c*_loss_	Cost distance-transform per-meter loss	−	10.00/15.00*	7.5
*c*_high_	High cost for cost transform	−	20.00	20.00
*c*_max_	Maximum cost for path planning	−	20.00	20.00
*σ*_sense_	Kalman filter cost measurement variance	−	0.10	0.10
$\sigma_{0}^{2}$	Kalman filter initial variance	−	1.00	1.00
*σ*_*s*_	GP output variance	−	1.00	1.00
*σ*_*ϵ*_	GP observation noise	−	0.50	0.50
*l*	GP length scale	−	0.40	0.40
$n_{L}^{\text{min}}$	Minimum learning set size	−	25.00	25.00
$n_{\text{erode}}^{\text{steps}}$	Cluster erosion steps	−	2.00	2.00
*m*_*T*_	Minimum size of a ground truth cluster	−	10.00	10.00
*d*_*S*_	Cost-model sampling lattice cell size	m	0.44	0.44
$n_{\text{dilate}}^{\text{steps}}$	Cluster dilation steps	−	3.00	3.00
$n_{\text{dilate}}^{\text{size}}$	Cluster dilation size	−	2.00	2.00
$\epsilon_{1}^{\text{IGNG}}$	GNG warp scale winner	−	1.00*e* − 3	1.00*e* − 3
$\epsilon_{\text{nb}}^{\text{IGNG}}$	GNG warp scale neighbor	−	1.00*e* − 5	1.00*e* − 5
$a_{\text{mature}}^{\text{IGNG}}$	GNG age mature	−	1.00*e*2	1.00*e*2
$a_{\text{max}}^{\text{IGNG}}$	GNG max edge age	−	50.00	50.00
*n* ^IGNG^	GNG learning batch size	−	5.00*e*3	5.00*e*3
Δ*t*_sample_	Cost sampling duration	s	30.00	12.00
Δ*t*_fallback_	Stuck fallback duration	s	30.00	3.00

* Different value used in *small* simulation/real deployment.

**TABLE 4:** System operation frequencies.

Module	Frequency	Condition
Elevation mapping	5.00 Hz	
Spatial goal identification	0.33 Hz	
Cost measurement	20.00 Hz	Only if using the respective gait
Cost learning	0.10 Hz	Only if not already running
Goal identification	0.10 Hz	
Goal sequence planning	1.00 Hz	Only after goal set change or reaching a goal
Path planning	1.00 Hz	Only after goal set change or reaching a goal

### 5.1 Simulated scenarios

**FIGURE 6:**
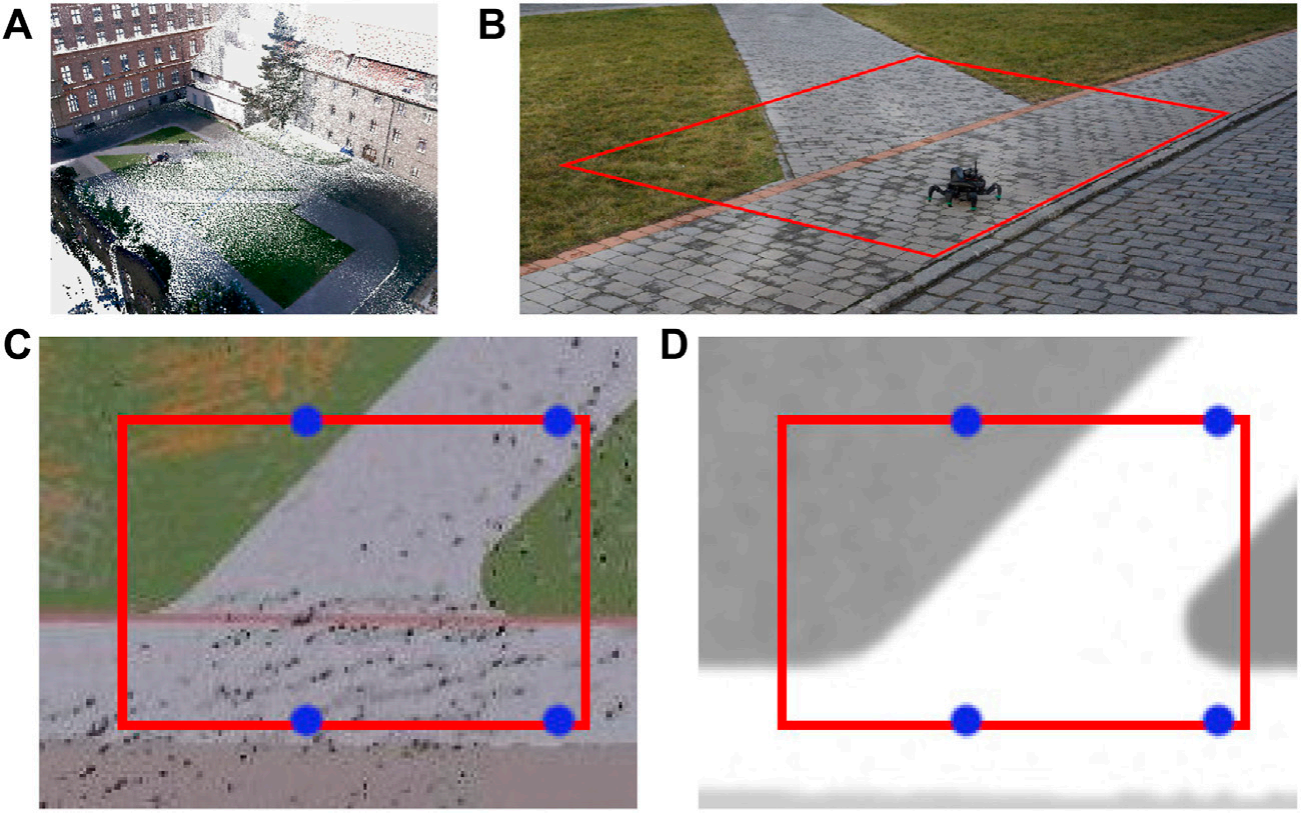
**(A)** 3D scan of the university campus at Charles Square in Prague, **(B)** section of the courtyard, and the respective simulated environment **(C)** color and **(D)** relative traversability (light areas easier to traverse). The red bounding box represents the area where the robot should explore. The blue points are the points to be visited by the robot in the first test tour.

The simulated scenarios are based on a courtyard environment captured by four 3D scans obtained using Leica BLK 360 3D scanner and visualized in Figure 6A. The scanner has standard deviation of 4 mm at 10 m and 7 mm at 20 m. The scans total approx. 1.4×10^8^ points.

In total, two virtual environments are created using the scan: small and large. The small environment represents a small section of the courtyard, where the simulated robot mimics the real robot’s speed and sensory equipment. It is used to test the benefit of the individual components of the proposed approach by comparing them to baseline methods where the particular component is removed or simplified. The large environment comprises terrain segments observed in the scan that are rearranged to create a larger, artificial environment with obstacles where different exploration algorithms are compared using a faster robot with an extended sensor range.

### 5.1.1 Small environment

The small environment is concerned with a section of the environment that is detailed in Figure 6B. We have created a simulation model of the environment containing several types of pavement (gray and red) and turf (green, brown), shown in Figure 6C. The turf is modeled as hard to traverse and can get the robot stuck for the fast gait, whereas the pavement does not impede the robot, see Figure 6D.

First, to demonstrate the benefits of using a cost model learned from prior experience, the robot is tasked to execute two tours in the environment using the learned cost model and a flat-cost baseline model. Second, the utility of exploring along the proposed GTSP-derived path is demonstrated by comparing its time to explore the environment with a greedy, myopic baseline, which drives the robot to the cheapest goal to reach w.r.t. the so far learned costs.

The first tour comprises four waypoints. The robot starts at the bottom-left point and executes the tour counter-clockwise until reaching the start location again. The two particular areas are designed to demonstrate the utility of the learned model: 1) the segment between the bottom-right and top-right waypoints where the robot can choose either a direct route over the turf or a longer path over the pavement and 2) the area around the top-left waypoint where the turf cannot be avoided and thus the robot needs to switch to the tall gait. The second tour comprises 20 points randomly sampled in the environment, and it serves to demonstrate the performance of the learned model over a tour that was not handcrafted.

In addition to the proposed approach and the baseline, in the simulated tests, we also deploy a hybrid gait selection approach that chooses its gait using the proposed model but does not plan its path w.r.t. the predicted costs and walks directly to the next waypoint. Unlike the baseline approach, which switches to the tall gait when stuck and repeatedly tries to switch back to the fast gait, the hybrid gait selection approach switches gaits only when approaching or leaving the terrain identified as hard to traverse by the model. Hence, it should outperform the baseline over longer sections on difficult terrains, where the baseline is slowed down by trying to switch back to the fast gait.

The simulation environment consists of the Intel i7-9700 3.00 GHz with 32 GB memory running Ubuntu 18.04 with ROS Melodic. Since the captured environment comprises terrains that might slow down the robot because they are somewhat non-rigid, instead of using a geometry-based simulator such as Gazebo, which cannot model such terrains, we elect to build a virtual environment over a simple simulator using real-world data. The simulation is performed using the simple two dimensional robot simulator (STDR)^3^ within the ROS ecosystem. On top of the simulator, we have implemented an interface that simulates the robot’s RGB-D camera, which assigns each point in the robot’s simulated exteroceptive measurements color based on the point’s position in the environment color map shown in Figure 6C and filters the measurements to contain only points within the 87 deg wide field of view of the simulated RGB-D camera. The terra-mechanical properties are simulated by slowing down the robot over the individual traversed terrains w.r.t. the performance observed over such terrain in a real-world deployment, as shown in Figure 6D.

**FIGURE 7:**
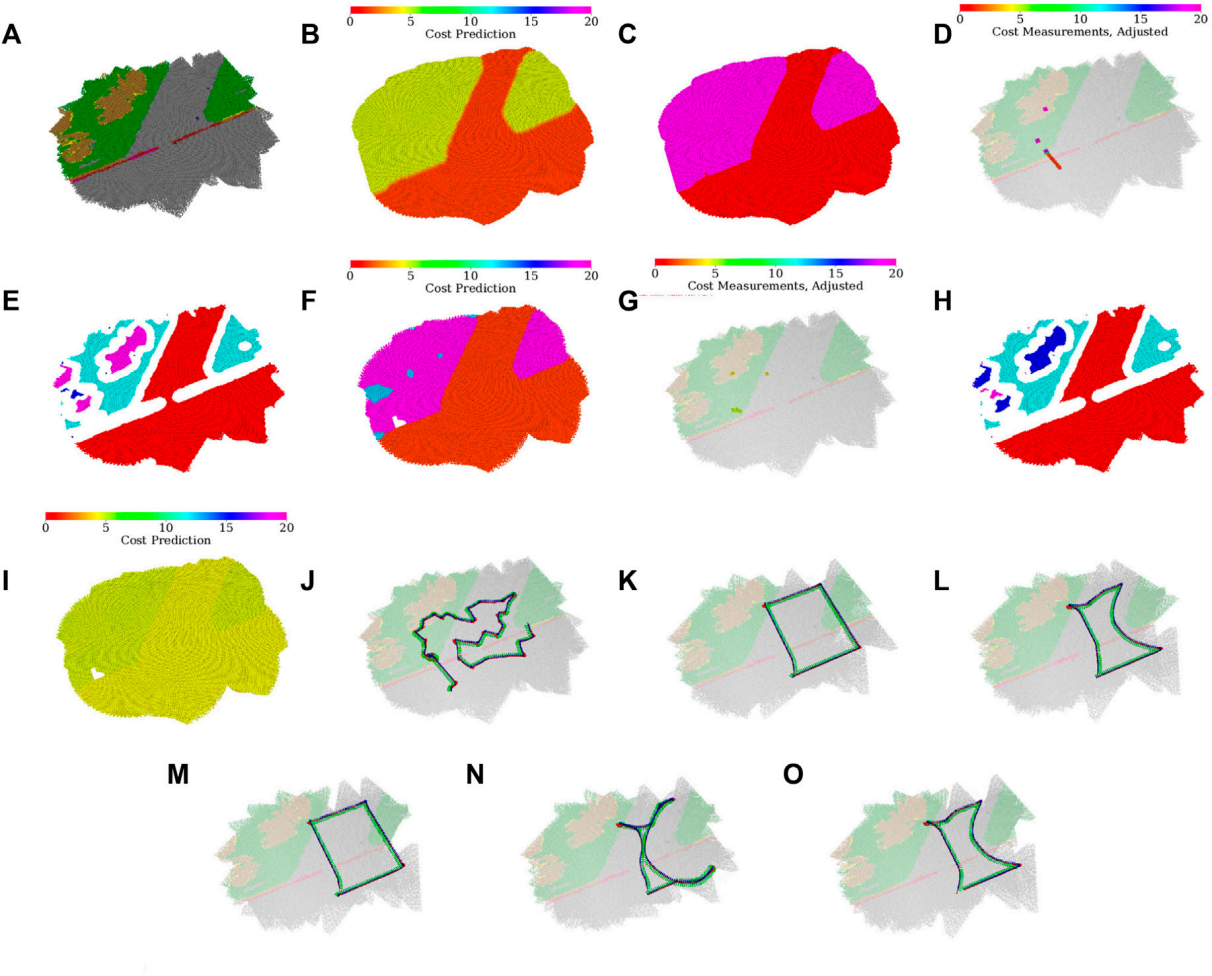
Environment assessment after the simulated scenario run with regards to both gaits; **(A)** dominant color in the histogram feature; **(B)** merged cost used for planning; **(C)** selected gait (fast in red, tall in purple); **(D)** costs used for learning the fast gait model [adjusted by hyperbolic tangent in (Eq. 20)], visualized over the terrain appearance; **(E)** clusters used in the fast gait model (arbitrary colors used to distinguish clusters); **(F)** fast gait cost predictions assigned by the dilated clusters; **(G)** costs used for learning the tall gait model [adjusted by hyperbolic tangent using (Eq. 20)], visualized over the terrain appearance; **(H)** clusters used in the tall gait model (arbitrary colors used to distinguish clusters); **(I)** tall gait cost predictions assigned by the dilated clusters; **(J)** exploration run; **(K)** test-tour run using the baseline model without the learned traversal costs; **(L)** test-tour run using the learned traversal costs. The development of the path through the fully discovered simulated environment during the exploration; **(M)** at the beginning of the exploration, the robot uses flat costs and thus does not avoid difficult terrains; **(N)** after learning the costs for the fast gait, the robot is too cautious and avoids going near the costly turf; **(O)** after learning the tall gait costs, the robot is less cautious and is willing to walk near difficult terrain.

In the evaluation, the robot first explores and learns the models shown in Figure 7A to Figure 7I. An example exploration path can be seen in Figure 7J. The robot learns that the turf, which appears either green or brown, cannot be traversed by the *fast* gait and thus selects the *tall* gait over that terrain type. On the other hand, the pavement does not hinder the *fast* gait, which is considerably faster and thus preferred.

Although the two gait models create the terrain clusters independently, the clusters in Figure 7E and Figure 7H differ only in cluster indices used in the internal representation (each index is associated with a different color in the visualization). It can be observed that the robot does not use any clusters associated with the red line on the pavement, either removing the thin cluster outright in the erosion or pruning the small erosion remains after the robot finds out that it cannot get enough samples to learn such a small terrain.

In the particular exploration run shown in Figure 7J, the robot first walks along the left side of the exploration bounds, learning the fast gait costs for both the pavement and turf and the tall gait cost over the turf. Then the robot learns the tall gait cost over the pavement while clearing the spatial exploration goals. During the exploration, it can be seen that the robot avoids walking over the remaining turf, only approaching it at the very end of the exploration. Thus, the robot needs only to enter and not leave the turf (minimizing the time on the costly terrain) to reach the goal that lies on the turf.

The test runs using the baseline, and the learned model over the first tour are shown in Figure 7K and Figure 7L, respectively. In addition, the development of the tours that would be used at different points during the exploration can be seen in Figure 7M through Figure 7O. In the baseline test, the robot walks directly between the waypoints and only switches to the *tall* gait after getting stuck. On the other hand, when using the learned model, the robot avoids the turf if possible and switches to the tall gait before entering the turf while pursuing the top-left goal.

The performance over 25 simulated trials (five exploration runs, each with five tour tests for the tour tests; 25 runs for the simulated exploration tests) can be observed in Table 5. On the first tour, the hybrid gait selection approach is slower than the reactive baseline. In the authors’ opinion, it is caused by the conservative (large) value of *r*_robot_, which compels the robot to use the slow tall gait on the border between the rough terrain and pavement, whereas the reactive approach only tries to switch back to the fast gait (which is its main disadvantage when compared to the hybrid approach) a few times on the short rough terrain segment. Nonetheless, the proposed learned model knows to avoid such areas and performs better or the same as the other approaches over every tour segment. Hence, the results suggest that robot benefits from using the learned costs in path planning. Over the second tour, the robot performs similarly. The learned model outperforms the baseline when moving around or over the turf. Both approaches exhibit similar travel times when the direct path between the waypoint leads only over the pavement. Unlike over the first tour, the hybrid gait selection performs better than the baseline approach, presumably due to longer sections over hard-to-traverse terrains on the second tour. The proposed approach consistently outperforms the baseline and hybrid gait selection approaches; we conclude that the robot benefits from using the learned model.

**TABLE 5:** Performance as the time (total cost) in seconds to traverse.

Small virtual environment, tour 1 (mean ± std of **25** runs)					
Method/Time [s]	Segment 1	Segment 2	Segment 3	Segment 4	Full tour
Baseline	79.99 ± 0.00	239.59 ± 6.62	133.20 ± 6.76	177.59 ± 13.04	630.39 ± 21.06
Gait selection	80.00 ± 0.00	275.00 ± 8.06	125.49 ± 7.39	164.00 ± 7.39	644.50 ± 7.34
Proposed	80.00 ± 0.00	119.99 ± 0.00	112.40 ± 4.27	142.40 ± 4.27	454.80 ± 4.27

**Small virtual environment, tour 2 (** **25** **runs)**			**Small virtual Environment, exploration (5 runs)**	
Method/Time [s]	Full tour		Environment	Time [s]	
Baseline	2748.00 ± 30.59		GTSP	1382.68 ± 241.47	
Gait selection	2523.12 ± 39.48		Greedy	1547.16 ± 203.71	
Proposed	2271.99 ± 33.38		

**Large virtual environment (mean ± std of ** **5** ** runs)**			**Real deployment**
Method	Full tour time [s]	Exploration time [s]	Test	Time [s]
Proposed	554.00 ± 13.56	1167.15 ± 163.69	Test Segment, baseline	454.00
Spatial-only	859.99 ± 156.02	545.40 ± 137.43	Test Segment, proposed	143.00
			Exploration, proposed*	1364.00

* The similarity between the real and simulated times to explore is coincidental.

In addition to the tour tests, the results suggest that the robot benefits from using the non-myopic GTSP planner compared to the myopic greedy approach. Even though the performance of the two approaches appears relatively close, the Mann–Whitney U Test (Mann and Whitney, 1947) rejects the null hypothesis of the same exploration time distribution at 99.5% confidence against both the two-sided and the relevant one-sided alternative. In the authors’ opinion, the high variance in the observed exploration times can be attributed to the effect of random chance in exploration since neither myopic nor non-myopic approaches are informed about the terrains in unexplored areas. However, the myopic explorer is more likely to make a bad decision, such as not clearing some of the goals in a particular area that needs to be visited later. Therefore, the proposed non-myopic approach performs better overall.

### 5.1.2 Large environment

**FIGURE 8:**
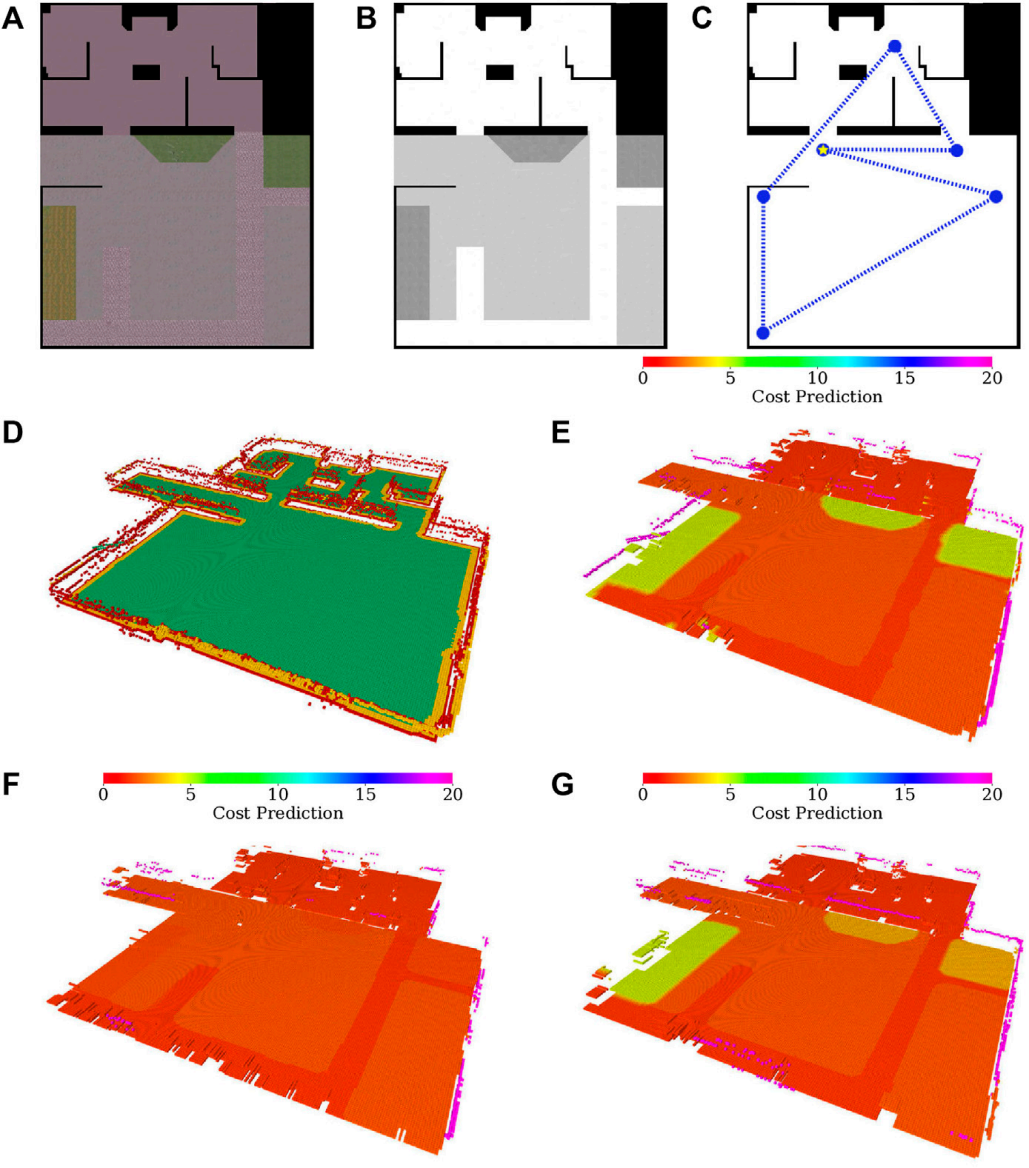
Large simulated environment **(A)** color and **(B)** relative traversability, **(C)** and the test tour through the environment, which starts at the starred node and is counter-clockwise. The built maps of the large simulated environment: **(D)** geometric map and **(E)** merged costs used for planning after exploration using the proposed approach; merged costs ofter exploration using the spatial-only model while **(F)** avoiding and **(G)** traversing rough terrain, respectively.

The large environment is an artificial 20 × 25 m outdoor/indoor scenario. The map comprises patches from the courtyard scan rearranged as shown in Figure 8. Given the size of the environment, the robot is sped up five times. The cell size is increased to 0.1 m, and other parameters are adjusted accordingly, see Table 3. In addition, the robot uses an omnidirectional sensor with the increased range of 10 m, which expands the range of terrains that can be observed without the respective terrain’s traversal. To accommodate the simulation of the increased sensor range, the virtual environment is run on AMD Ryzen Threadripper 3960× 3.8 GHz with 48 GB memory running Ubuntu 18.04 and ROS Melodic, using STDR in the same manner as for the small environment.

Similar to the small environment, the robot is first set to explore the environment and then is tasked to visit the set of waypoints shown in Figure 8C. The proposed algorithm is compared to a spatial-only baseline approach, which learns the cost models only as a result of experiencing cost while pursuing spatial exploration goals. The spatial-only changes the gaits in a reactive fashion when stuck and hence only learns the model for the tall gait if it enters the difficult green or brown turf during the exploration.

The quantitative results for the large environment are shown in Table 5. Since the proposed approach actively tries to sample every terrain type, it is slower to explore the whole environment. However, the proposed approach performs better in the tour evaluation. Closer examination suggests that while the tour times of the proposed approach remain similar in all trials, the spatial-only times vary wildly since the learned models differ based on which terrains the robot has traversed during the exploration. This randomness can be attributed to differences in simulation and plan execution. In addition, Figures 8D–G shows the learned maps for the proposed model, and for the spatial-only model in both the cases when the rough terrain was and was not traversed. For the case when a rough terrain was traversed by the spatial-only model, the costs differ between the individual rough areas. However, the ground truth costs shown in Figure 8B suggest that they should be the same, as is the case for the proposed model. Likely, this is caused by the robot traversing only the brown-green rough terrain located on the left of the environment. The green terrain, located in the center and right of the environment, appears somewhat similar to the brown-green terrain. Hence, the robot considers it to be difficult to traverse to a certain degree. However, since the spatial-only model does not deliberately sample the terrains, the model’s guess differs somewhat from the exact cost to traverse the particular terrain, decreasing the fidelity of the predictions.

Overall, the presented results suggest that the proposed approach presents a tradeoff in terms of exploration and execution time: the longer time spent exploring the environment and learning the cost models provides the robot with better cost maps, which shorten the time to navigate the environment after it is explored. It should be noted that since the behavior of the spatial-only model is affected by random chance (differences in simulation and plan execution), it can provide models as good as the proposed approach. However, there is no guarantee that this would happen regularly, whereas the proposed approach has returned high fidelity maps in every test case.

### 5.2 Real robot experimental deployment

**FIGURE 9:**
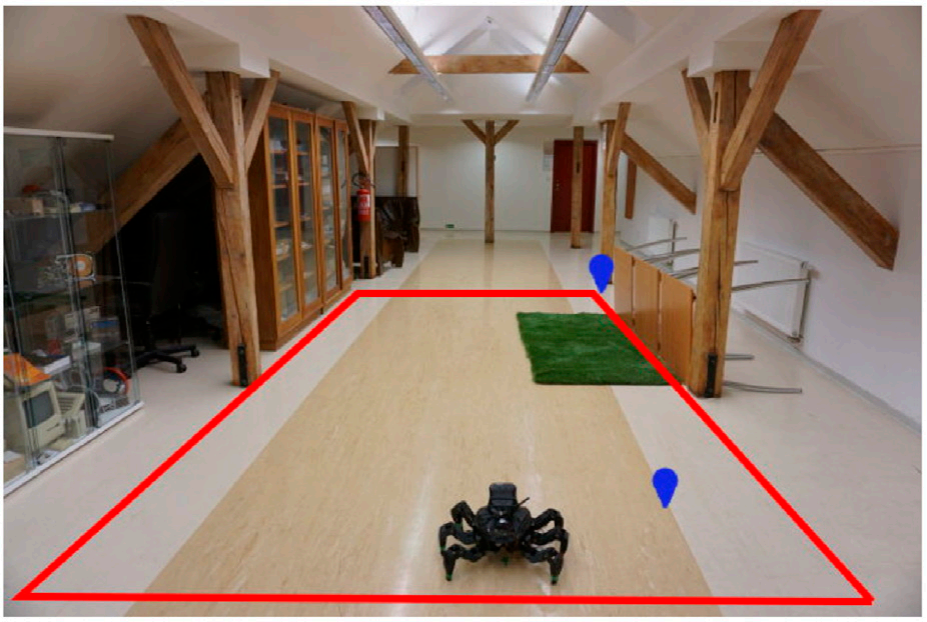
The 2m × 6m large deployment area with a green artificial turf. The area boundary is in red, and the waypoints of the test tour are depicted in blue. The shown robot is at the starting position.

The viability of the proposed approach is demonstrated in the real experimental deployment, where the robot explores an indoor 2 × 6 m area visualized in Figure 9. The office-like environment comprises flat synthetic terrain that is easy to traverse but appears to the robot differently colored at different locations since it is glossy and carries the color of nearby objects located next to the arena. When building the colored elevation map $M_{2.5D}$ , we use the first color observed at each location to account for the issue. In addition to the flat terrain, a green artificial turf is placed in a part of the test area to provide a relatively hard terrain to traverse. The robot interacts with the real terrains similarly to the simulation: the *fast* gait may get stuck on the turf but is faster than the *tall* gait over the flat parts of the arena. During the experiment, the robot is set to explore the area; even though it can leave the bounds of the 2 m × 6 m large area, it does not pursue goals located outside of the bounds.

**FIGURE 10:**
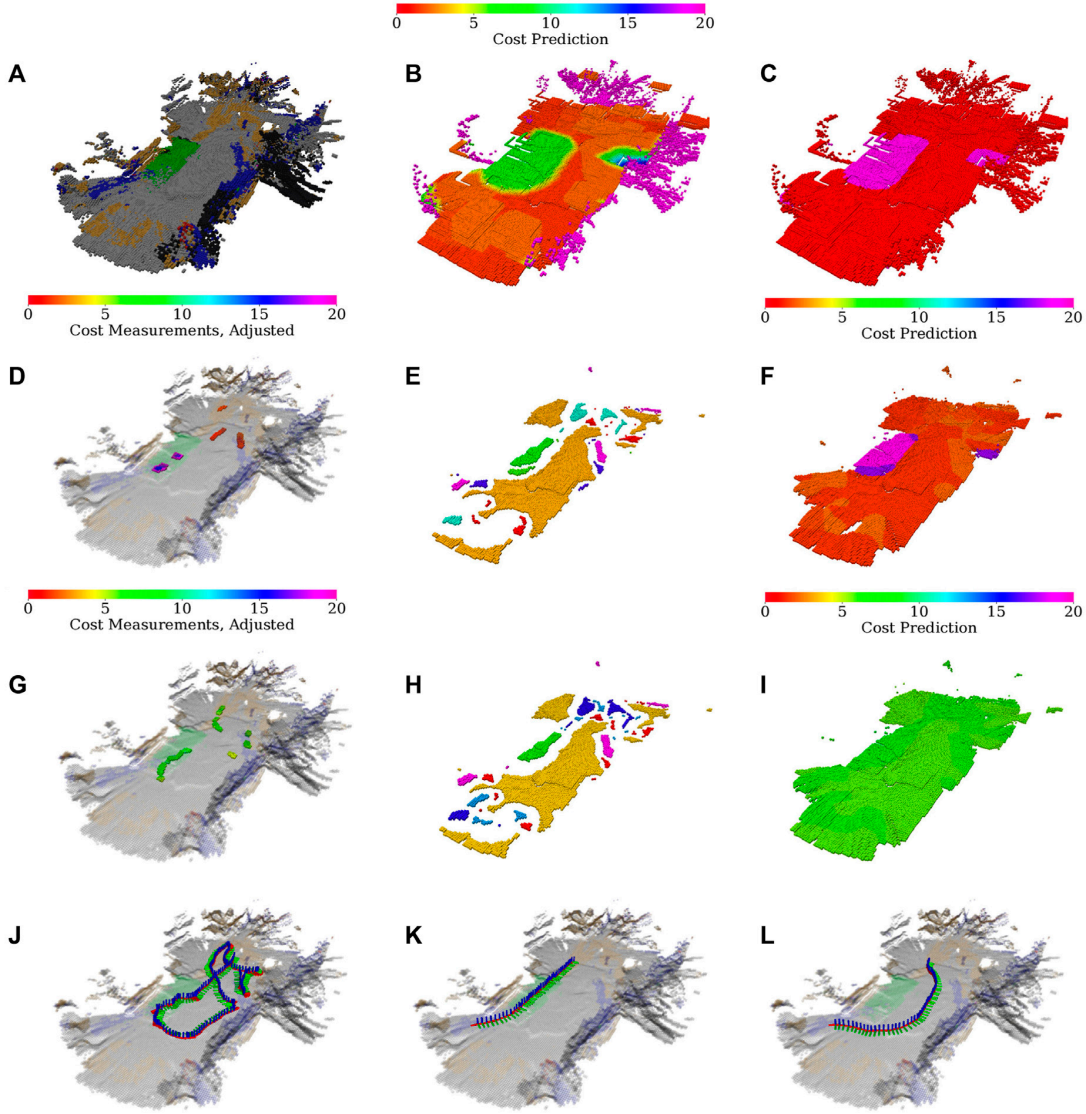
Environment evaluation and the real robot exploration run; **(A)** dominant color in the histogram feature; **(B)** merged cost used for planning; **(C)** selected gait (*fast* in red, *tall* in purple); **(D)** costs used for learning the *fast* gait model (adjusted by hyperbolic tangents), visualized over the terrain appearance; **(E)** clusters used in the fast gait model (arbitrary colors used to distinguish clusters); **(F)** fast gait cost predictions assigned by the dilated clusters; **(G)** costs used for learning the tall gait model (adjusted by hyperbolic tangents), visualized over the terrain appearance; **(H)** clusters used in the tall gait model (arbitrary colors used to distinguish clusters); **(I)** tall gait cost predictions assigned by the dilated clusters; **(J)** exploration run; **(K)** test-tour run using the baseline model without the learned traversal costs; **(L)** test-tour run using the learned traversal costs.

Figure 10 shows the maps learned in the experimental run, which is also presented in the accompanying Supplementary Video S1. A colored map of the environment is depicted in Figure 10A. The overall costs and selected gaits through the environment are shown in Figure 10B and Figure 10C, respectively.

During the experimental deployment, the robot first learns the largest gray appearing flat terrain using the fast gait. Then it learns on the turf for both gaits and returns to the gray area to learn for the tall gait. Afterward, the robot pursues the yet unvisited spatial goals and smaller off-color terrain clusters that appear near the environment boundary and are caused by the glossy floor that carries the color of the nearby objects.

Compared to the simulation, the robot needs a larger amount of the measurements to learn the terrains (see Figure 10D and Figure 10G), and there are more terrain clusters (see Figure 10E and Figure 10H). It suggests that the real environment is noisier and contains multiple differently colored areas, which is in line with our observations regarding the glossy floor material. Nevertheless, the traversal costs learned by the robot for the individual gaits (see Figure 10F and Figure 10I) are within expectations, as is the overall planning cost depicted in Figure 10B and gait selection visualized in Figure 10C.

The test run scenarios are set up similarly to the tours used in the simulated test; the robot is placed in front of the hard-to-traverse turf and tasked to reach a goal location behind the hard-to-traverse terrain, slightly out of the exploration bounds, see Figure 9. The paths shown in Figure 10K and Figure 10L show that when using the baseline without the learned model, the robot tries to reach the goal directly over the turf, gets stuck, and needs to switch to the slow *tall* gait. On the other hand, when using the learned model, the robot avoids the hard-to-traverse areas and reaches its goal quickly using the fast gait. The performance in the presented run can be seen in Table 5. Overall, we conclude that the real deployment confirms that the robot can actively learn the traversability as a part of the exploration mission and benefits from using such learned models.

## 6 Discussion

The presented exploration system is proposed as a combination of spatial geometric modeling and learning terrain-gait traversal cost models. However, the system is designed to support additional models that do not describe the robot’s traversal cost. Moreover, since the models are kept separate, there is no need to use the same feature set for each of them. Therefore, the approach is compatible with spatial models such as magnetism models (Karolj et al., 2020) or GP-based occupancy (Wang and Englot, 2016). The only requirement for a model is that it produces a set of learning goals in the environment that are resolved once particular information is sampled. Hence, the proposed system can be extended by including additional traversability models, such as modeling the passability of potentially non-rigid obstacles.

In addition, we approach the traversal cost prediction so that it supports any cost model that is additive along the traversed path, such as time to traverse or consumed energy. Besides, individual cost predictors describe the gaits of a hexapod walking robot, but they can also describe any discrete set of robot configurations. Hence, the approach is viable for any mobile robot that describes its motion experience using an additive cost and can also be used to model the energy a tracked robot consumes, for example, with adjustable flippers. A particular limitation of the cost modeling used in the presented approach is that we assume that the individual gaits are switched for free w.r.t. the cost (i.e., instantaneously for cost modeled as the time to traverse), whereas in practice, the gait requires some time to exhibit its properties. In this study, we leave the question of how to predict gait-change cost open for future work.

The used cost model goal generation stems from the idea that adding new observations does not increase GP uncertainty if the hyper-parameters are fixed (Rasmussen and Williams, 2006). Therefore, sampling new measurements should not increase uncertainty and thus not spawn new goals in areas containing none. In practice, even though we use fixed GP hyper-parameters, the non-increasing nature of the uncertainty does not strictly hold for the approximated information gain since, in addition to the GP hyper-parameters, the information gain also depends on the terrain clusters and the costs and descriptors in the learning set, all of which might drift during the exploration. However, the robot behavior demonstrated in both evaluation setups shown in Figure 7J and Figure 10J suggests that the assumption holds in general. The robot clears the areas corresponding to the individual terrains (goals) and is not compelled to return to previously visited areas.

The primary limitation of the proposed approach is identified in its inability to compare the utility of the goals originating from the different models. We are motivated to build a modular system that would support different model types; therefore, the proposed decoupled approach considers each goal equally valued, regardless of the source model. This limits how the models are used since the goal utility, such as the information gain, is relegated to be used only inside the particular model to determine which environment features (locations or terrain types) are goals to use in creating an instance of the GTSP. The proposed approach provides a non-myopic solution to visit the goals reported by the individual models, where the models are also non-myopic since each can report multiple goals. Myopic models that would report their respective highest utility goal (potentially with multiple sampling sites) can be used. However, similarly to the myopic planner with the results reported in Table 5, the time to explore would likely increase since the GTSP planner would lack the information on where to go after the current goals are sampled, and thus the exploration path would often change significantly. Integrating goal utility into the decoupled planning and using alternative utility functions such as the GP-UCB remains the subject of future work.

## 7 Conclusion

In this study, we present a system for autonomous mobile robot exploration that incorporates active learning of traversal cost models in addition to spatial model building. During the exploration, the robot builds the spatial geometric model of the environment and learns the traversal cost models, each comprising a Gaussian process regressor and a growing neural gas terrain clustering scheme. The geometric model is used to determine areas passable by the robot, while the cost models predict the traversal costs over the passable terrains from the terrain’s appearance. Each cost model corresponds to a particular hexapod walking robot locomotion gait. The robot approaches exploration in a decoupled manner, creating a set of goals for the spatial exploration and for each traversal cost model. The exploration path is planned by solving an instance of the generalized traveling salesman problem over the goals that are sets of possible sites of visits to improve the particular model. The proposed system has been evaluated in simulation setup and real experimental deployment with two different walking gaits. The results suggest that the proposed system yields the robot to explore the environment and learn the traversal cost models. The learned models benefit the robot’s operation in the environment. In future work, we plan to model the gait change costs, include additional traversability models such as obstacle rigidity, and extend the proposed approach to support goal utility and exploration–exploitation models.

## Data Availability

The raw data supporting the conclusion of this article will be made available by the authors, without undue reservation.

## Footnotes

1 In the simulated experiments, the localization is provided by the simulator.

2 In practice, for small environments, it is feasible to compute the prediction for every cell, and we do so for visualization as depicted in Figure 5G and Figure 5H.

3
http://stdr-simulator-ros-pkg.github.io
