# Supplementary material for: Autonomous robotic exploration with simultaneous environment and traversability models learning
Source: Front Robot AI. 2022 Oct 5;9:910113. doi: 10.3389/frobt.2022.910113 (PMC9581159; doi:10.3389/frobt.2022.910113)
Supplement: Supplementary file 1 [file DataSheet1.pdf]

SUPPLEMENTARY APPENDIX S1

TERRAIN CLUSTER EROSION AND DILATION

In practice, it is not desirable to place cost exploration goals at the boundaries of terrains classes because, in such areas, a real robot with the imprecise path following might fail to traverse the correct terrain, and the descriptors in such areas might be distant from the prototype $\mathrm{ta}(T)$. Besides, it might not be possible to acquire enough samples to learn the traversal cost on a small terrain area of a particular class. Hence, after assigning the terrain classes to cells, we erode cells that border different (or already eroded) terrain class using

$$T^{--}(\nu) = \begin{cases} T^{-}(\nu) & \text{if } \forall \nu' \in \mathrm{8nb}(\nu) : T^{-}(\nu) = T^{-}(\nu'), \\ \varnothing & \text{otherwise,} \end{cases} \tag{1}$$

where $\varnothing$ is the eroded non-class terrain, T^{-} and T^{--} are the class assignments before and after an erosion step, respectively, and the erosion process is repeated $n_{\text{erode}}^{\text{steps}}$-times.

As a result of the erosion, some cells are assigned the eroded non-class $\varnothing$ with no prototype to use. Hence, when assigning cost predictions for path planning, we first dilate the terrain classes by selecting the most common class in the vicinity as

$$T^{++}(\nu) = \begin{cases} \mathrm{argmax}_{T \in \mathcal{T}} \sum_{\nu' \in \mathrm{8nb}^{n_{\text{dilate}}^{\text{size}}}(\nu)} |T = T^{+}(\nu')| & \text{if } \exists \nu' \in \mathrm{8nb}^{n_{\text{dilate}}^{\text{size}}}(\nu) : T^{+}(\nu') \neq \varnothing, \\ \varnothing & \text{otherwise,} \end{cases} \tag{2}$$

where $\mathrm{8nb}^{n_{\text{dilate}}^{\text{size}}}$ is the $n_{\text{dilate}}^{\text{size}}$-times repeated neighborhood function $\mathrm{8nb}$, T^{+} and T^{++} are the class assignments before and after a dilation step, respectively, and the dilation process is repeated $n_{\text{dilate}}^{\text{steps}}$-times.

SUPPLEMENTARY APPENDIX S2

GAUSSIAN PROCESS REGRESSION

Assuming function $f(x)$ that is observed with the noise ϵ

$$y = f(x) + \epsilon, \quad \epsilon \in \mathcal{N}(0, \sigma_\epsilon^2), \tag{3}$$

Gaussian Process (GP) is defined as the distribution

$$f(x) \sim \mathcal{GP}(m(x), K(x, x')), \tag{4}$$

where $m(x)$ is the mean

$$m(x) = E\left[f(x)\right], \tag{5}$$

and $K(x, x')$ is the covariance

$$K(x, x') = E\left[\left(f(x) - m(x)\right)\left(f(x') - m(x')\right)\right]. \tag{6}$$

Given the training data X, the GP regressor's predictions and the query X_* are

$$\begin{aligned} \mu(X_*) &= K(X, X_*)\left[K(X, X) + \sigma_\epsilon^2 I\right]^{-1} y, \\ (\sigma(X_*))^2 &= K(X_*, X_*) \\ &\quad - K(X, X_*)^T\left[K(X, X) + \sigma_\epsilon^2 I\right]^{-1} K(X, X_*), \end{aligned} \tag{7}$$

where $K(X, X')$ is the covariance function.

SUPPLEMENTARY APPENDIX S3

INCREMENTAL GROWING NEURAL GAS

The *Incremental Growing Neural Gas* (IGNG) is a soft-computing clustering approach proposed by Prudent and Ennaji (2005). The approach builds on the *Growing Neural Gas* (GNG) (Fritzke, 1994), which adapts a graph topology to continually provided measurements. However, unlike the GNG, which is enlarged after a fixed number of measurement adaptation steps, the IGNG is only grown when adapting to a value x that is out of the bounds of the current structure.

Algorithm 1: Incremental Growing Neural Gas: Adaptation

Input: Ω – IGNG structure with terrain classes $\mathcal{T}$; x – Adapted measurement for the terrain descriptor $\mathtt{ta}$.

Output: Ω – IGNG structure for the terrain classes $\mathcal{T}$).

Procedure `adaptIGNG`(Ω, x)
- $\omega_1 \leftarrow \mathrm{argmin}_{\omega \in \Omega_{\text{neurons}}} \|x, \omega\|$ // Find the closest neuron to the adapted measurement.
- $\omega_2 \leftarrow \mathrm{argmin}_{\omega \in \Omega_{\text{neurons}}/\omega_1} \|x, \omega\|$ // Find the second closest.
- **if** $|\Omega_{neurons}| = 0 \vee \|x, \omega_1\| > \sigma^{IGNG}$ **then** // If there are no neurons or the closest is too far.
  - $\Omega_{\text{neurons}} \leftarrow \Omega \cup \omega_{\text{new}}, \omega_{\text{new}} = x$ // Add the measurement as a new neuron.
- **else**
  - **if** $|\Omega_{neurons}| = 1 \vee \|x, \omega_2\| > \sigma^{IGNG}$ **then** // If there is only 1 neuron or the second closest is too far.
    - $\Omega_{\text{neurons}} \leftarrow \Omega_{\text{neurons}} \cup \omega_{\text{new}}, \omega_{\text{new}} = x$ // Add the measurement as a new neuron.
    - $\Omega_{\text{connections}} \leftarrow \Omega_{\text{connections}} \cup (x, \omega_1)$ // Connect the new neuron with the closest.
  - **else**
    - $\omega_1 \leftarrow \omega_1 + \epsilon_1^{\text{IGNG}}(x - \omega_1)$ // Warp the closest neuron to the measurement.
    - **for** $\omega_{nb} \in nb(\omega_1)$ **do** // For each neighbor of the closest neuron.
      - $\omega_{\text{nb}} \leftarrow \omega_{\text{nb}} + \epsilon_{\text{nb}}^{\text{IGNG}}(x - \omega_{\text{nb}})$ // Warp it to the measurement.
      - $a(\omega_1, \omega_{\text{nb}}) \leftarrow a(\omega_1, \omega_{\text{nb}}) + 1$ // And age their connections.
    - **if** $(\omega_1, \omega_2) \in \Omega_{connections}$ **then** // If the first and closest are connect.
      - $a((\omega_1, \omega_2)) \leftarrow 0$ // Reset the connection age.
    - **else**
      - $\Omega_{\text{connections}} \leftarrow \Omega_{\text{connections}} \cup (\omega_1, \omega_2)$ // Otherwise insert new connection.
    - **for** $\omega_{nb} \in nb(\omega_1)$ **do** // For each neighbor of the closest neuron.
      - $a(\omega_{\text{nb}}) \leftarrow a(\omega_{\text{nb}}) + 1$ // Age the neighbor.
- **for** $(\omega_a, \omega_b) \in \Omega_{connections} : a((\omega_a, \omega_b)) > a_{max}^{IGNG}$ **do** // Find too old connections.
  - $\Omega_{\text{connections}} \leftarrow \Omega_{\text{connections}}/(\omega_a, \omega_b)$ // And remove them.
- **for** $\omega \in \Omega_{neurons} : a(\omega) \geq a_{mature}^{IGNG}$ **do** // Find isolated mature neurons.
  - **if** $\neg \exists \omega' \Omega_{neurons} : (\omega, \omega') \in \Omega_{connections}$ **then** // And remove them.
    - $\Omega_{\text{neurons}} \leftarrow \Omega_{\text{neurons}}/\omega$
- **return** Ω

The IGNG adaptation is summarized in Alg. 1, and it operates as follows[1]. The algorithm keeps a graph of neurons (graph vertices) and their connections (graph edges) and keeps an age value for each neuron and connection. When adapting to a new measurement x, the algorithm first finds the closest neuron ω_1 and the second closest neuron ω_2. If the graph is empty or the closest neuron is too far with $\|x - \omega_1\| > \sigma^{\text{IGNG}}$, a new embryo neuron ω_{new} with the age $a(\omega_{\text{new}}) = 1$ is inserted at x. If ω_1 is close enough, but the second closest ω_2 is not, or there is only one neuron in the graph, a new neuron is also inserted at x. Moreover, an edge between the new neuron and ω_1 is inserted into the graph with the age $a((\omega_1, \omega_{\text{new}})) = 0$.

If both ω_1 and ω_2 are close enough, ω_1 and all of its neighbors (neurons with an existing connection to ω_1) are warped towards x by ϵ_1^{IGNG} and $\epsilon_{\text{nb}}^{\text{IGNG}}$, respectively. Then, if there is already a connection between ω_1 and ω_2, its age is set to 0. Otherwise, the connection is created. Next, the ages of all neighbors $a(\omega_{\text{nb}})$ of ω_1 and their respective connections $a((\omega_1, \omega_{\text{nb}}))$ are incremented by one.

After adapting to the measurement, the graph is pruned to remove old connections and isolated neurons. In general, it is desirable for neurons to be old (since measurements were often observed near then) and for connections to be young (since measurements were recently observed along the edge). First, we identify neurons that are mature with $a(\omega) \geq a_{\text{mature}}^{\text{IGNG}}$. Then, connections that are too old with $a((\omega, \omega')) > a_{\text{max}}^{\text{IGNG}}$ are removed from the graph. If it leads to isolated mature neurons, these are also removed.

REFERENCES

Fritzke, B. (1994). A growing neural gas network learns topologies. In *Conference on Neural Information Processing Systems (NIPS)*. 625–632

Prudent, Y. and Ennaji, A. (2005). An incremental growing neural gas learns topologies. In *International Joint Conference on Neural Networks (IJCNN)*. vol. 2, 1211–1216. doi:10.1109/IJCNN.2005.1556026

[1] The herein presented description is limited to the basic operation of the algorithm and omits its use for semi-supervised labeling since it is not used in the presented work. We refer the interested reader to Prudent and Ennaji (2005).
